# *In Silico* Identification of Chiral Biflavonoids as Dual PI3Kα/mTOR Inhibitors

**DOI:** 10.1021/acsomega.5c06196

**Published:** 2025-09-19

**Authors:** Francisca Fernanda Nunes Azevedo, Francisca Joseli Freitas de Sousa, Jonatas Martins Negreiro, Jaqueline Vieira Carletti, Maria Conceição Ferreira Oliveira, Geancarlo Zanatta

**Affiliations:** † Postgraduate Programme in Biochemistry, Department of Biochemistry at Federal University of Ceará, Fortaleza 60440-554, CE, Brazil; ‡ Department of Biochemistry, Federal University of Rio Grande do Sul, Porto Alegre 90035-003, RS, Brazil; § Department of Organic and Inorganic Chemistry, Science Center, Federal University of Ceará, Fortaleza 60455-760, CE, Brazil; ∥ Postgraduate Programme in Cellular and Molecular Biology at Federal University of Rio Grande do Sul, Porto Alegre 90650-001, RS, Brazil

## Abstract

The PI3K/AKT/mTOR pathway is crucial in regulating key processes in mammalian cells, and impairments of this pathway are associated with cell survival in several cancer types. PI3Kα is the second most mutated oncogenic protein, and its overactivation initiates a secondary signaling cascade that enhances, among others, the activity of mTOR complexes 1 and 2. Despite the importance of this pathway, there is a gap in identifying dual inhibitors targeting both PI3Kα and mTOR, which could potentially overcome the limitations of single-target therapies. In this study, advanced computational tools were employed to identify plant-derived compounds with selective or dual inhibitory potentials against PI3Kα and mTOR enzymes. Compounds were obtained from the NuBBe database, a catalogue of Brazilian natural compounds. Among the 1745 compounds docked against the PI3Kα and mTOR enzymes, four bioflavonoids (2–5) displaying atropisomerism stood out. These compounds were further investigated for their binding profile into the catalytic sites of PI3Kα and mTOR, considering the influence of their sense of chirality (*Ra* and *Sa* enantiomers). The results indicate that compound 2 had no enantiopreference for PI3Kα, while (*Sa*)-2 preferentially bound to mTOR. Compound 3 bound to mTOR in both configurations, while only (*Ra*)-3 bound to PI3Kα. Compound 4 showed no enantiomeric preference for either protein, whereas compounds (*Ra*)-5 bound to PI3Kα and (*Sa*)-5 bound to mTOR. Altogether, these findings highlight the potential of four novel bioflavonoid compounds exhibiting a sense of chirality as promising candidates for the rational design of new cancer therapeutics targeting PI3Kα and mTOR. These insights provide a robust foundation for developing potent dual inhibitors, offering new avenues for treating diseases associated with the hyperactivation of these enzymes. Furthermore, this research underscores the value of plant-derived natural products in developing effective therapeutic agents, contributing significantly to the field of medicinal plant research and advancing the frontier of medicinal chemistry.

## Graphical abstract

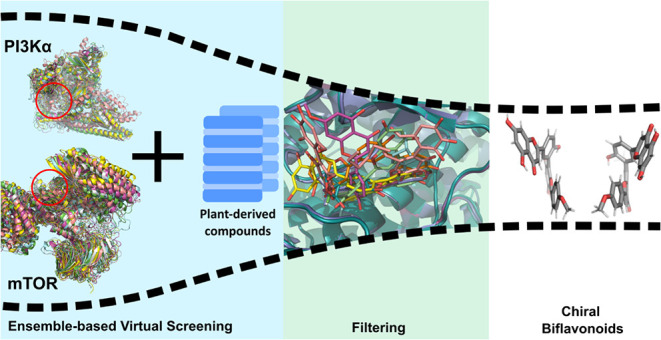

## 1 Introduction

The phosphoinositide 3-kinase (PI3K)/AKT/mTOR pathway plays a critical role in regulating key metabolic processes, including cell proliferation, survival, and growth.^1^ Aberrant activation of this pathway is implicated in numerous cancers, occurring in approximately 70% of breast and ovarian cancers, 90% of lung adenocarcinomas (ADCs), and 40% of squamous cell carcinomas (SCCs).^2,3^

In recent decades, significant efforts have focused on developing PI3Ks/AKT/mTOR-targeting drugs for anticancer therapy.^4,5^ These efforts have led to the emergence of various classes of inhibitors, including selective, pan, and dual PI3K inhibitors,^6–10^ selective mTOR inhibitors^11^ and dual PI3K/mTOR inhibitors.^9,12–14^

One of the primary advantages of using multitarget inhibitors within this pathway is the potential reduction in toxicity. Targeting multiple enzymes with a single inhibitor may allow for lower dosages, potentially reducing the toxicity. Moreover, sustained inhibition associated with multitarget inhibitors may reduce the likelihood of resistance development, as they act at multiple points along the same pathway.^15,16^

Among the sources of molecular scaffolds for the development of new drugs, natural products are highlighted as a rich source of secondary metabolites displaying a large spectrum of biological activities, including cytotoxicity against human tumor cells.^17,18^ Flavonoids are reported as one of the major chemical classes of bioactive natural products.^19,20^ These compounds are mainly found in fruits and vegetables and have shown promising inflammatory and cytotoxic activities in the treatment of chronic diseases, including cancer.^21^

Biflavonoids, a subclass of flavonoids, have also shown important pharmacological properties, such as anti-inflammatory, anticancer, antiviral, antimicrobial, and antithrombotic activity by acting in the PI3K/AKT/mTOR pathway.^17,22,23^ Boosting a dimeric flavonoid structure, bioflavonoids are characterized by restricted rotation around their interflavonoid bond, leading to atropisomerism^24^ and resulting in stable enantiomers designated as *Ra* or *Sa*.^25^ This stereochemical feature is particularly relevant for multitarget inhibition strategies, as individual enantiomers may enhance dual-target selectivity and efficacy while potentially reducing the required dosage and minimizing off-target toxicity.^26^ In contrast, racemic mixtures (equimolar *Ra*/*Sa*) can introduce variability in molecular interactions, potentially compromising both the selectivity and pharmacodynamic consistency.

Given the potential advantages of enantioselective multitarget inhibitors in reducing toxicity and enhancing therapeutic efficacy, biflavonoids emerge as promising candidates for the dual inhibition of PI3K and mTOR. Despite their pharmacological potential, flavonoids face well-documented challenges in drug development, including limited target selectivity, complex structure–activity relationships, poor bioavailability, and metabolic instability frequently linked to cytochrome P450 interactions. These hurdles have historically limited their clinical advancement. In this context, recent advances in computational methodologies facilitate systematic evaluation of binding selectivity, interaction stability, and pharmacokinetic properties of identified compounds, enabling the prioritization of natural biflavonoids with favorable drug-like profiles. Accordingly, this study employs an integrated approach combining ensemble-based virtual screening, molecular dynamics simulations, and MM-PBSA free-energy calculations to identify biflavonoids with a dual PI3K/mTOR inhibitory potential. This workflow supports the selection of compounds with robust interaction profiles and provides structural insights that may guide future optimization toward multitarget activity and improved pharmacokinetics.

To achieve this, we employed an ensemble-based virtual screening protocol to assess the binding potential of 1745 plant-derived compounds from the NuBBE database to the ATP-binding sites of PI3Kα and mTOR. Promising hits were subjected to molecular dynamics simulations, followed by MM-PBSA calculations to estimate binding free energies and decompose the energetic contributions of key interacting residues. This integrative strategy identified biflavonoid scaffolds with potential as enantioselective dual inhibitors of PI3K/mTOR, offering a pathway toward improved therapeutic profiles.

## 2 Results and Discussion

In this work, four biflavonoid molecules displaying dual PI3K/mTOR inhibitory behavior were identified among 1745 phytochemicals from the Brazilian database (NuBBE). To avoid bias from experimental geometries during virtual screening, the docking of each compound was simulated against a conformational ensemble. After the top-score compounds showing atropisomeric properties were identified, the enantiopreference of each of them was evaluated through ensemble docking followed by molecular dynamics simulations. Finally, their binding geometries and interaction profiles were described using free-energy calculations.

### 2.1 Ensemble Generation

One of the main pitfalls when performing virtual screening simulations against biological targets is underestimating the degree of freedom such targets might present.^27^ Indeed, using only a single-target structure in semiflexible docking approaches very often leads to inconsistent affinity ranking, as only ligands with similar shapes and sizes of the rigid binding pocket tend to show good affinity. To overcome these limitations, ensemble-based virtual screening employs conformational ensembles of target proteins, thus exploring the conformational space of target pockets during the screening and enabling the selection of more accurate binding geometries.^28^ In this study, self- and cross-docking were used to select, among available experimental coordinates, a set of PI3Kα and mTOR structures capable of reproducing the experimental geometries of the ligands. As shown in Figure S1 (Supporting Information), better self-docking results for mTOR were obtained when using a search area of dimensions 20 × 20 × 20 (*x*,*y*,*z*), while no major differences were observed for PI3Kα using the same dimensions or a larger box (30 × 30 × 30). For clarity and for the sake of reproducibility, Figure S7 shows the location of the selected docking region employed in this study.

Cross-docking simulations identified the smallest set of experimental structural targets required to recover the experimental geometries of the ligands. Using a search area of 20 × 20 × 20 for both proteins, we selected structures with the smallest deviation in ligand binding geometries compared to experimental crystallographic data. For PI3Kα, nine crystallographic ligands were docked into each of the nine crystallographic structures. As shown in Figure S2 (Supporting Information), the PI3Kα structure 5XGI reproduced the crystallographic geometries of six out of the nine ligands (with RMSD values below 3Å), while 4YKN reproduced the geometries of seven of the nine ligands. Additionally, 4L23 was included, as it represents the only experimental data available for a dual PI3K/mTOR inhibitor. To further improve the conformational ensemble of PI3Kα structures, we added 3HIZ and 4LIB, as these structures represent the protein in its free state (without a ligand). For mTOR, cross-docking simulations revealed that structure 4JSV reproduced the geometry of five out of six ligands, while 4JSX reproduced four ligands and 4JSP and 3JBZ reproduced three ligands each. Structures 4JT5 and 4JT6 reproduced only two ligands each (Figure S3, Supporting Information). Additionally, 4JSN was included in the final conformational ensemble, as it represents a receptor in its free state. Both ensembles were used during the ensemble-based virtual screening simulations.

The observation that distinct PI3Kα and mTOR conformers preferentially reproduce different subsets of ligands supports the well-established notion that ensemble-based docking enhances pose prediction accuracy and reduces false negatives by accounting for binding-site plasticity.^54^ Moreover, the inclusion of apo structures is consistent with evidence that unliganded states may provide access to alternative binding-site geometries relevant for ligand accommodation.^55^ Nonetheless, while ensemble-based docking improves robustness over single-structure approaches, it remains limited to experimentally available conformations and cannot fully describe the protein’s dynamic landscape.

### 2.2 Ensemble-Based Virtual Screening

A total of 1,745 phytochemical compounds from the NuBBE database were docked against conformational ensembles of both PI3Kα and mTOR. The top 10% scoring compounds (Supporting Table S2) were further analyzed to assess their pharmacokinetic properties and potential for dual inhibition. From this subset, four biflavonoid compounds (labeled 2 to 5) were prioritized based on their binding scores and chemical relevance. These biflavonoids demonstrated enantioselective binding, suggesting the potential for dual PI3K/mTOR inhibition via atropisomeric configurations.

To further evaluate the drug-likeness of the selected biflavonoids, their ADME (absorption, distribution, metabolism, and excretion) profiles were assessed using the SwissADME platform.^44^ As shown in the Supporting Table 16, compounds 2, 4, and 5 exhibited only one violation of Lipinski’s Rule of Five, specifically a log *P* value exceeding 5, while meeting the criteria for molecular weight and the number of hydrogen bond donors and acceptors.^52^ In contrast, compound 3 presented two violations related to its higher molecular mass and an excessive number of hydrogen bond donors. However, such deviations are commonly observed among natural products and do not necessarily compromise the bioavailability, particularly when absorption occurs through active transport mechanisms. According to the expanded interpretation proposed by Newman and Cragg,^53^ which recognizes the relevance of transporter-mediated uptake, these biflavonoids remain promising candidates for further investigation in the drug discovery pipeline.

The planar chemical structures of compounds 2 to 5 are depicted in Figure 1, with each distinct chemical moiety color-coded for clarity. Compound **2** (7″,4″’-dimethoxy-amentoflavone) is formed by a methoxyphenyl moiety region (i), connected to a 7-methoxy-2-methyl-2,3-dihydro-4H-chromen-4-one moiety region (ii), which is linked to a hydroxyphenyl moiety region (iii), that connects to a noreugenin moiety region (iv). Compound **3** (amentoflavone) is formed by a hydroxyphenyl moiety region (i), connected to a noreugenin moiety region (ii), which is linked to another hydroxyphenyl moiety region (iii), connected to a noreugenin moiety region (iv). Compound **4** (heveaflavone) comprises a methoxyphenyl moiety region (i), connected to a 7-methoxy-2-methyl-2,3-dihydro-4*H*-chromen-4-one moiety region (ii), which is linked to a hydroxyphenyl moiety region (iii) connected to another 7-methoxy-2-methyl-2,3-dihydro-4*H*-chromen-4-one moiety region (iv). The last identified biflavonoid compound is compound **5** (podocarpusflavone A). This compound is formed by a methoxyphenyl moiety region (i), connected to a noreugenin moiety region (ii), which is linked to a hydroxyphenyl moiety region (iii) that connects to another noreugenin moiety region (iv).

**Figure 1:**
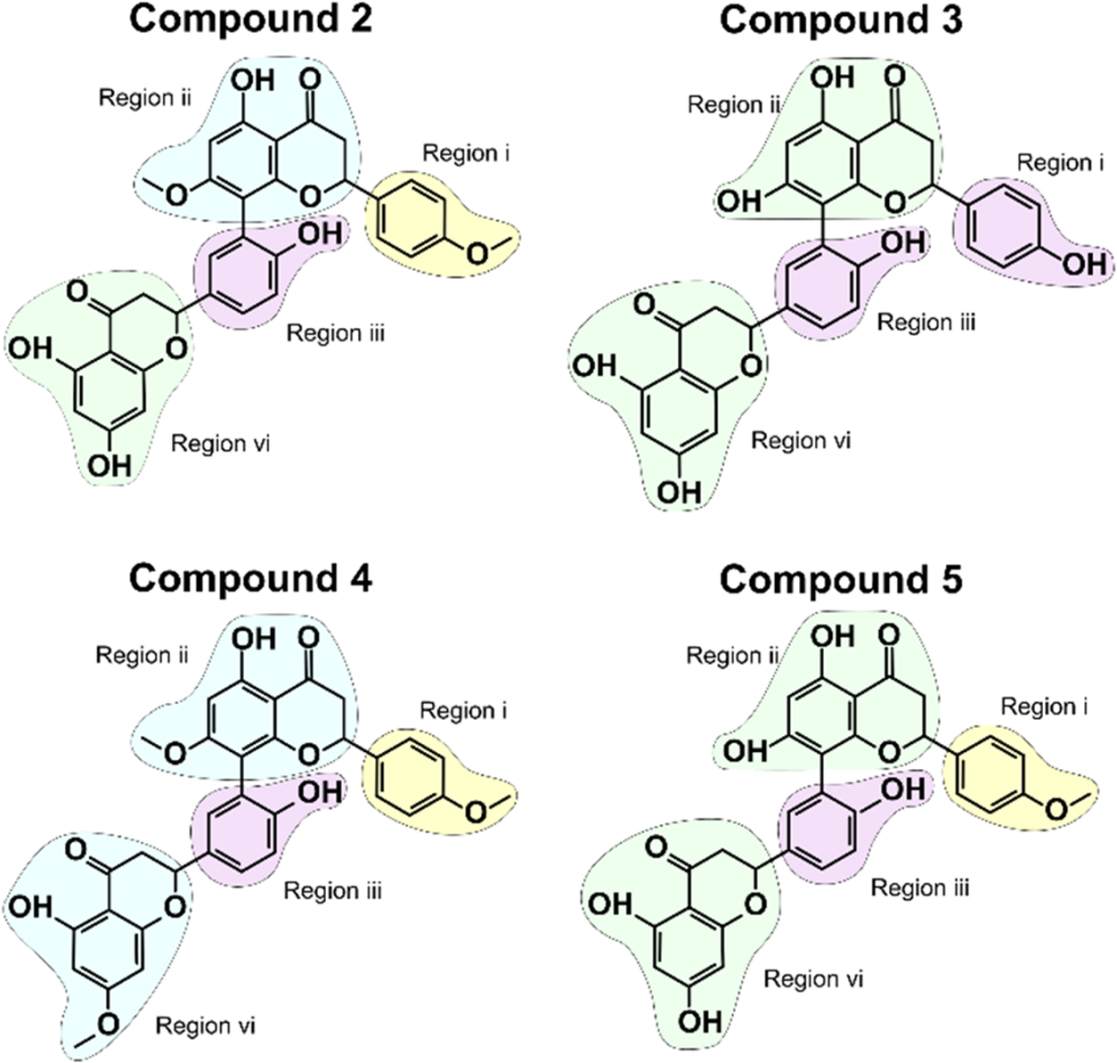
Chemical representation of biflavonoid compounds (2–5). Regions are labeled (i–iv) and colored according to their structural similarity. Importantly, all four biflavonoid compounds contain an atropisomeric center in the axial rotation in the bond between region (ii) and region (iii).

Docking results revealed that both atropisomers ((*Ra*)- and (*Sa*)-2) of compound 2 are capable of binding to PI3Kα; however, only the (*Sa*)-2 configuration demonstrated preferential binding to mTOR. Conversely, both enantiomers of compound 3 exhibited favorable binding to mTOR, whereas only the (*Ra*)-3 configuration showed an affinity for PI3Kα. Interestingly, compound 4 did not display a pronounced enantiomeric preference, with both (*Ra*) and (*Sa*) forms binding comparably to PI3Kα and mTOR. For compound 5, enantioselectivity was more distinct: the (*Ra*)-5 isomer preferentially bound to PI3Kα, while (*Sa*)-5 exhibited stronger interactions with mTOR. A summary of the predicted enantiomeric binding preferences, as determined through ensemble docking simulations, is provided in Table 1

**Table 1:** Preferential Binding of Compounds 2–5 According to Their Configuration (*Sa* or *Ra*)

	PI3Kα		mTOR	
	input *Sa*	input *Ra*	input *Sa*	input *Ra*
#	output	output	output	output
2	*Sa*	*Ra*	*Sa*	*Sa*
3	*Ra*	*Ra*	*Sa*	*Ra*
4	*Sa*	*Ra*	*Sa*	*Ra*
5	*Ra*	*Ra*	*Sa*	*Sa*

### 2.3 Atropisomeric Compounds: Binding Profile Analysis

To better characterize the binding of the atropisomeric compounds **2–5**, each protein–ligand complex, considering the relevant configuration (*Sa*, *Ra*, or both), was subjected to induced fitting simulations through molecular dynamics (MD) followed by binding energy analysis through MM-PBSA calculations.

Figure S4 (Supporting Information) shows the RMSD trajectories of the complexes over 200 ns of MD simulation. All systems reached equilibrium within the first 20 ns, except for the mTOR­(*Sa*)-2 complex, which stabilized after approximately 40 ns, and the PI3Kα­(*Ra*)-3 complex, which equilibrated around 80 ns. These exceptions may reflect ligand-induced conformational changes or enhanced flexibility within the binding site.

Although our primary analysis focused on the 200 ns simulations, to evaluate local flexibility at the protein–ligand interface and provide a comparative view along the MD simulations, we analyzed the root-mean-square fluctuation (RMSF) of binding-site residues over both 100 and 200 ns simulations (Supporting Figures S5 and S6). Overall, RMSF profiles remained consistent between both durations, suggesting that the structural dynamics of the complexes were stable throughout. Notably, PI3Kα­(*Sa*)-4 and PI3Kα­(*Ra*)-2 complexes exhibited nearly identical RMSF patterns at 100 and 200 ns, reinforcing the stability of the protein–ligand interactions. Conversely, PI3Kα­(*Ra*)-3 showed marginally increased fluctuations in a few residues at 200 ns, although these changes were not indicative of significant conformational drift.

For MM/PBSA calculations, representative snapshots were extracted from two separate segments of the 200 ns trajectories: one after system equilibration (90–100 ns) and another at the final segment (190–200 ns), as presented in Supporting Table S17. The similarity of MM/PBSA binding energies between these two intervals confirms that the systems had reached convergence before 90 ns. Therefore, the binding energy values reported in this study were computed from the final segment (190–200 ns) using 100 evenly spaced snapshots. The single-trajectory approach was employed to speed up calculations while delivering reliable results, and the SASA model was applied to evaluate per-residue contributions within each complex. Calculations were set up in accordance with a previous study,^27^ which fine-tuned parameters for PI3Kα and mTOR proteins. The absolute binding energy calculated with MM-PBSA is summarized in Table 2, and energy component details are depicted in Supporting Table S3.

**Table 2:** Top-Ranked Compounds Targeting PI3Kα or mTOR Proteins^a^

compound	PI3Kα	mTOR
(*Sa*)-**2**	–7.9	–14.9
(*Ra*)-**2**	–9.9	
(*Sa*)-**3**		–11.2
(*Ra*)-**3**	–14.6	–11.0
(*Sa*)-**4**	–13.4	–12.0
(*Ra*)-**4**	–11.7	–11.5
(*Sa*)-**5**		–15.6
(*Ra*)-**5**	–11.0	
Alpelisib	–12.4	
Torin 2		–9.11
PI103	–10.54	–13.35

a Results represent the MM-PBSA calculated energies, expressed in kcal/mol.

To investigate the interaction between the selected ligands and the target proteins, we analyzed the fluctuation of the interaction energy considering residues located at radial distances up to 10Å. For this purpose, the radial distance of each residue from the nearest atom of the ligand was measured, and the individual interaction energy was calculated and added up in 0.5Å increments. To avoid losing relevant residues, the convergence was considered when the total energy varied by less than 10% over a 2Å. Such an approach has been well established in previous publications and circumvents the use of arbitrary thresholds as it accounts for the stability of energy fluctuations along the distance from the binding pocket. Within this range, the residues that consistently exhibited the strongest attractive contributions across the different ligand configurations were primarily responsible for the slope observed between 2.0 and 4.5 Å, as observed in Figure 2. Notably, PI3Kα residues within 2.5 Å were the most important to stabilize the binding of compounds (*Ra*)-**4** and (*Sa*)-**2**, while residues within 3.5 Å were necessary to stabilize the binding of compounds (*Ra*)-**2**, (*Ra*)-**3**, and (*Sa*)-**4**, and 4.5 Å were necessary to stabilize compound (*Ra*)-**5**. Interestingly, based on the binding energies with PI3Kα, compounds (*Ra*)-**3** and (*Sa*)-**4** showed affinities comparable to or even higher than the selective inhibitor alpelisib, standing out as the most promising candidates. Compounds (*Ra*)-**2**, (*Ra*)-**4**, and (*Ra*)-**5** shared a partial overlap in key interacting residues with the dual-inhibitor PI103, in addition to showing comparable predicted binding affinities. PI103 is a well-characterized and potent dual PI3K-mTOR inhibitor, and its binding profile reflects this dual specificity. This partial similarity suggests that these ligands may display a less selective mode of action, although not necessarily reproducing the complete binding profile of PI103. In contrast, (*Sa*)-**2** displayed a weaker interaction pattern with reduced overlap in key residues.

**Figure 2:**
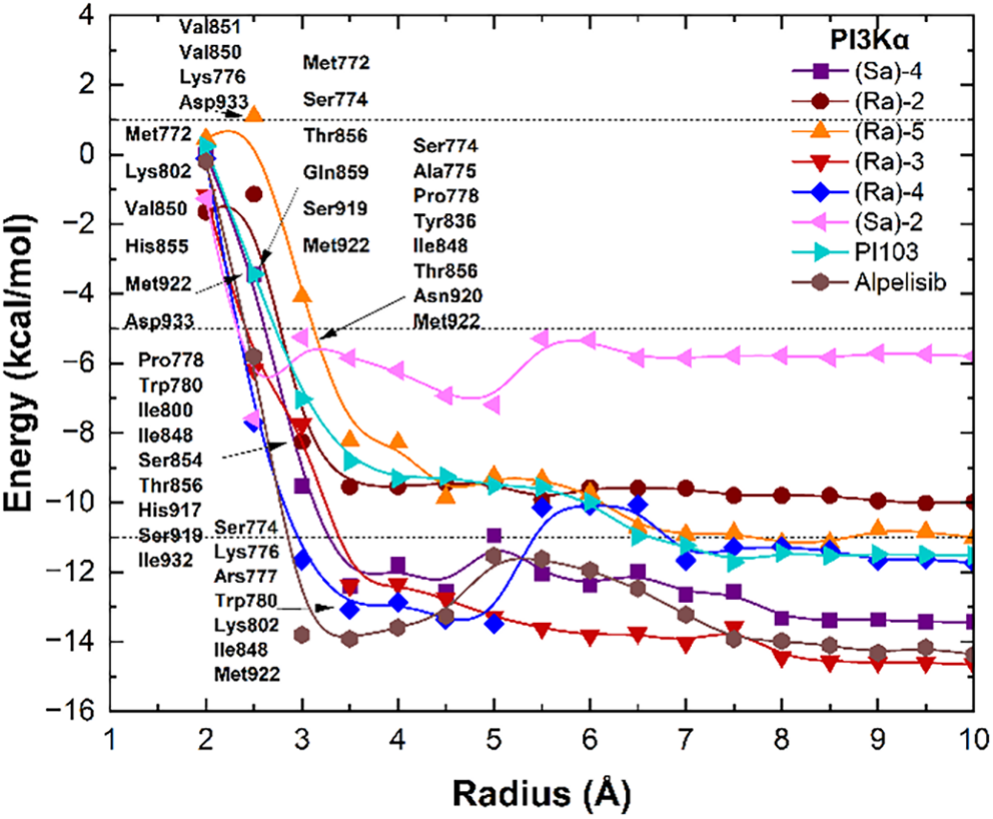
Fluctuation of the total interaction energy in PI3Kα complexes. Interaction energy is expressed in kcal/mol, and distance is expressed in angstroms.

As observed in Figure 3, residues Ile932, Ile848, Ile800, Val850, Trp780, Met772, and Met922 were identified as the main contributors to attractive interactions with all configurations. These residues are consistent with those reported in previous studies, which highlighted the same interaction with known inhibitors such as Alpelisib and PI103.^29–31^ The calculated contributions of all residues are displayed in Tables S4–S9 (Supporting Information). A visual inspection, as shown in Figure 4, shows that configuration (*Ra*)-**2** forms strong hydrophobic interactions with Ile800 and Ile932 through its aromatic rings in region ii and region i, respectively. Residue Trp780 forms a T-shaped π–π interaction with the aromatic ring in region ii, while on the other face of the cleft, residue Met922 is responsible for π–sulfur interactions with the same aromatic ring in region ii. Sitting next to Trp780, residue Met722 interacts with the aromatic ring in region iii through π–sulfur interactions. Such interactions between methionine residues and aromatic moieties seem to be present in one-third of all known protein structures in the Protein Data Bank.^32^ In addition, interactions between Met772 and Met922 have been previously observed in the binding stabilization of PI3K ligands.^33^ While Val850 forms hydrophobic interactions with region ii, two hydrogen bonds are formed between the hydroxyl and ketone substituents in region ii and the carboxyl and amine group terminals of Val851, helping stabilize (*Ra*)-**2** at the bottom of the pocket. Further stabilization is achieved through a strong interaction with residue Asp933. Specifically, a weak hydrogen bond between the hydroxyl group in region iv and the carboxyl group of Asp933 side chain holds the ligand within the pocket, while a repulsive interaction between the carboxyl group of Asp933 and the methoxy group in region i helps anchor the ligand to the bottom of the binding site. Configuration (*Sa*)-**2** (Figure 4B) forms a hydrogen bond between the hydroxyl group in region IV and Val851. In the hydrophobic region of the pocket, residues Ile800 and Ile848 interact with region i, while Ile932 interacts with aromatic rings in regions i and iv. Residue Trp780 forms a T-shaped π–π interaction with the aromatic ring in region iv. Residue Met772 interacts with aromatic rings in regions i and iii through π–sulfur interactions, while on the other face of the cleft, residue Met922 is responsible for π–sulfur interactions with the aromatic ring in region vi. In addition, Asp933 pushes the oxygen in the methoxy group in region (i) toward the bottom of the binding pocket. Configuration (*Ra*)-**3** sits in a hydrophobic pocket, where residue Ile932 interacts with regions ii and iii, Ile848 interacts with region iii, and Ile800 interacts with region iv. The hydroxyl group of region ii forms a strong hydrogen bond with the nitrogen atom in the peptide bond between Val850 and Val851. Trp780 forms a T-shaped π–π interaction with the aromatic ring in region I, while Met922 forms a π–sulfur interaction with the aromatic ring in region ii and Met772 forms a π–sulfur interaction with the aromatic ring in region iv. Configuration (*Sa*)-**4** (Figure 4D) forms hydrophobic interactions through the ring in region ii and residues Ile800, Ile848, and Ile932. Residue Trp780 forms a T-shaped π–π interaction with the aromatic ring in region i, while on the other face of the cleft, residue Met922 forms a π–sulfur interaction with the same aromatic ring. Residue Met772 forms a π–sulfur interaction with the aromatic ring in region iii. Asp933 repels the oxygen atom in region iv and the oxygen atom in the methoxy group of region ii. In addition, the hydroxyl group in region ii forms a hydrogen bond with the carboxyl group in the peptide bond between Glu849 and Val850. During the stabilization of the interaction of configuration (*Ra*)-**4** (Figure 4E), the oxygen atom of the methoxy group in region iv forms a hydrogen bond with the nitrogen atom in the peptide bond between Val850 and Val851. Residues Ile800, Ile848, and Ile932 hold hydrophobic interactions with rings in region iv. Residue Trp780 forms a T-shaped π–π interaction with the aromatic ring in region i. Residue Met922 forms two π–sulfur interactions, one with the aromatic ring in region i and another with the aromatic ring in region iv. Residue Met772 forms a π–sulfur interaction with the aromatic ring in region iii. When interacting with PI3Kα, configuration (*Ra*)-**5** (Figure 4F) is surrounded by residues Ile800, Ile848, and Ile932, which form hydrophobic interactions with aromatic rings in regions ii and iii. In addition, each hydroxyl group in region ii forms a hydrogen bond with the carboxy and amino terminals in the peptide bonds among Glu849-Val850-Val851. Residue Trp780 forms a T-shaped π–π interaction with the aromatic ring in region I, while Met922 forms a π–sulfur interaction with the same ring. Residue Met772 forms a π–sulfur interaction with the aromatic ring in region vi. Asp933 repels (*Ra*)-**5**, but with less intensity than observed for other compounds.

**Figure 3:**
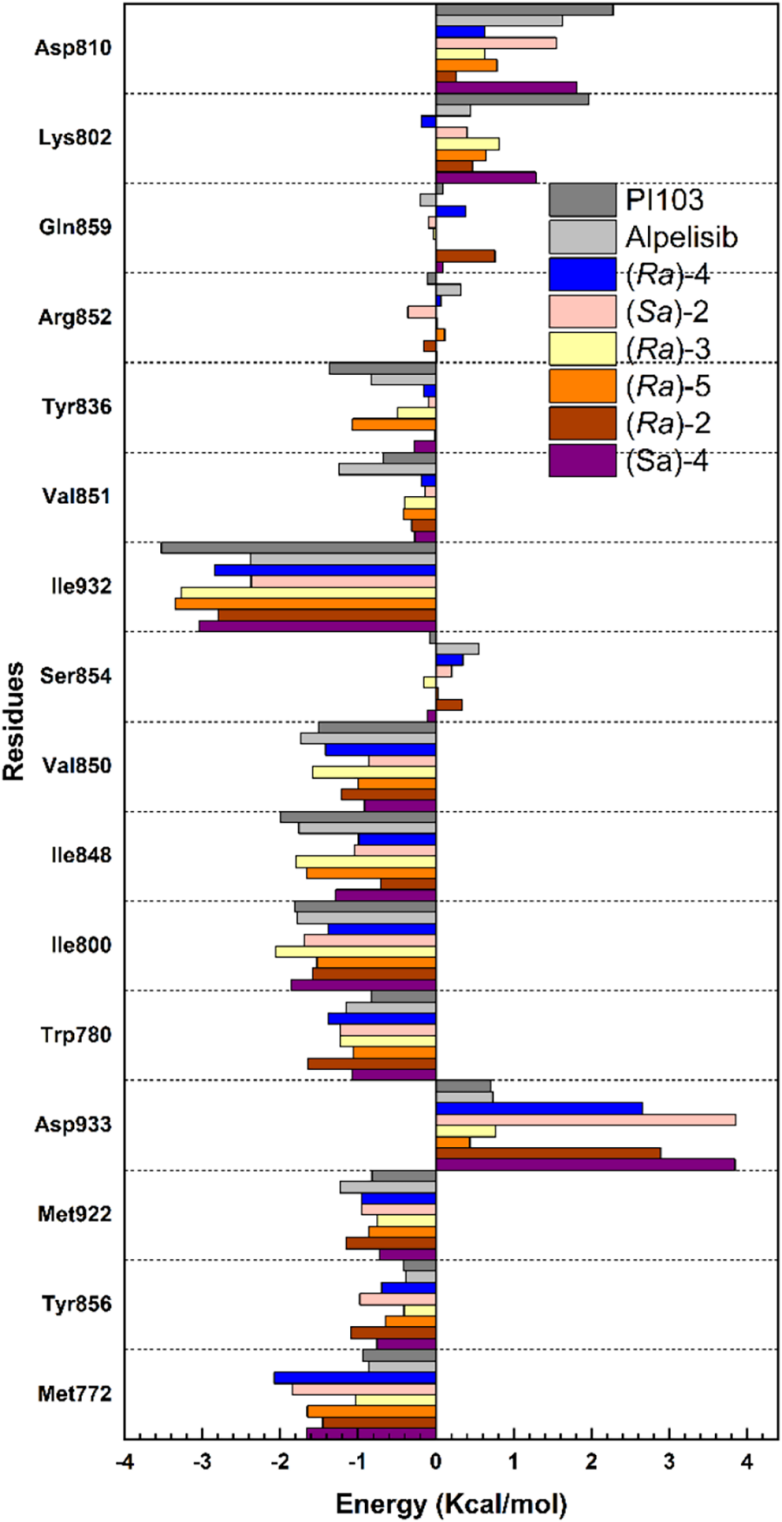
Individual interaction energy of residues at the ATP-binding site of PI3Kα.

**Figure 4:**
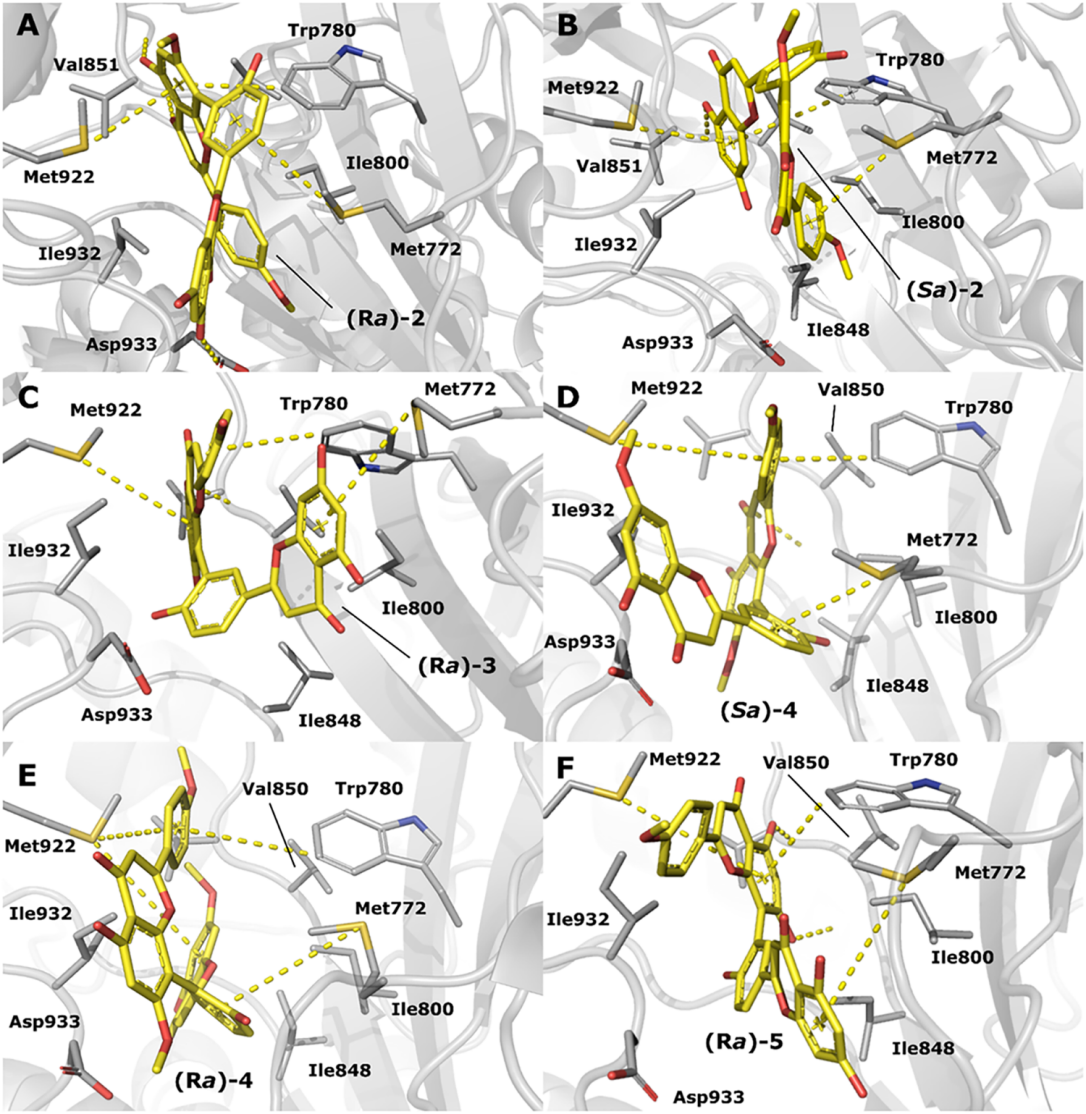
Graphical representation of compounds 2–5 bound to the ATP-binding site of PI3Kα, showing (A) (*Ra*)-**2** interactions; (B) (*Sa*)-**2** interactions; (C) (*Ra*)-**3** interactions; (D) (*Sa*)-**4** interactions; (E) (*Ra*)-**4** interactions; and (F) (*Ra*)-**5** interactions. Protein structures are illustrated as cartoons, while the residue side chains and ligands are represented as sticks.

In the next step of our analysis, the binding energy fluctuation of compounds **2–5** bound to mTOR was also analyzed. As depicted in Table 2, compounds (*Sa*)-**5** and (*Sa*)-**2** exhibited the strongest binding affinities to mTOR, outperforming dual-inhibitor PI103. Compounds (*Ra*)-**4**, (*Sa*)-**4**, and (*Ra*)-**5** also demonstrated favorable interactions, while compounds (*Sa*)-**3** and PI103 displayed lower binding affinities, indicating a comparatively weaker stabilization within the mTOR binding site. As shown in Figure 5, the binding energy profile of (*Sa*)-**2** displays a sharp decrease between 2.0 and 3.5 Å, followed by smooth stabilization at around 6.5 Å. Similarly, (*Sa*)-**5** shows a strong energy drop from 2.0 to 3.0 Å and gradually stabilizes up to 7.5 Å. For (*Sa*)-**4**, the curve presents a pronounced slope from 2.0 Å, reaching stability near 3.5 Å, with only minor fluctuations up to 10 Å. Configuration (*Sa*)-**3**, on the other hand, starts with a positive energy between 2.0 and 3.0 Å and then shows a steady and large decrease until stabilization close to 7.0 Å. The profile of (*Ra*)-**3** shows an initial slope up to 3.0 Å, remains steady until about 4.5 Å, and then decreases further with stabilization at 6.5 Å. Configuration (*Ra*)-**4** exhibits a decrease up to 4.5 Å, followed by mild fluctuations up to 9.0 Å before stabilizing. For Torin 2, a steep slope is observed between 2.0 and 3.0 Å, followed by positive fluctuations up to 7.5 Å. Notably, compounds (*Sa*)-**2**, (*Sa*)-**5**, and (*Sa*)-**4** maintained stable and favorable binding energies from 8 to 10 Å, showing interaction profiles comparable to or even more stable than Torin 2 and PI103. Compounds (*Sa*)-**3**, (*Ra*)-**3**, and (*Ra*)-**4** presented fluctuations in their binding energy profile with energy values similar to Torin 2.

**Figure 5:**
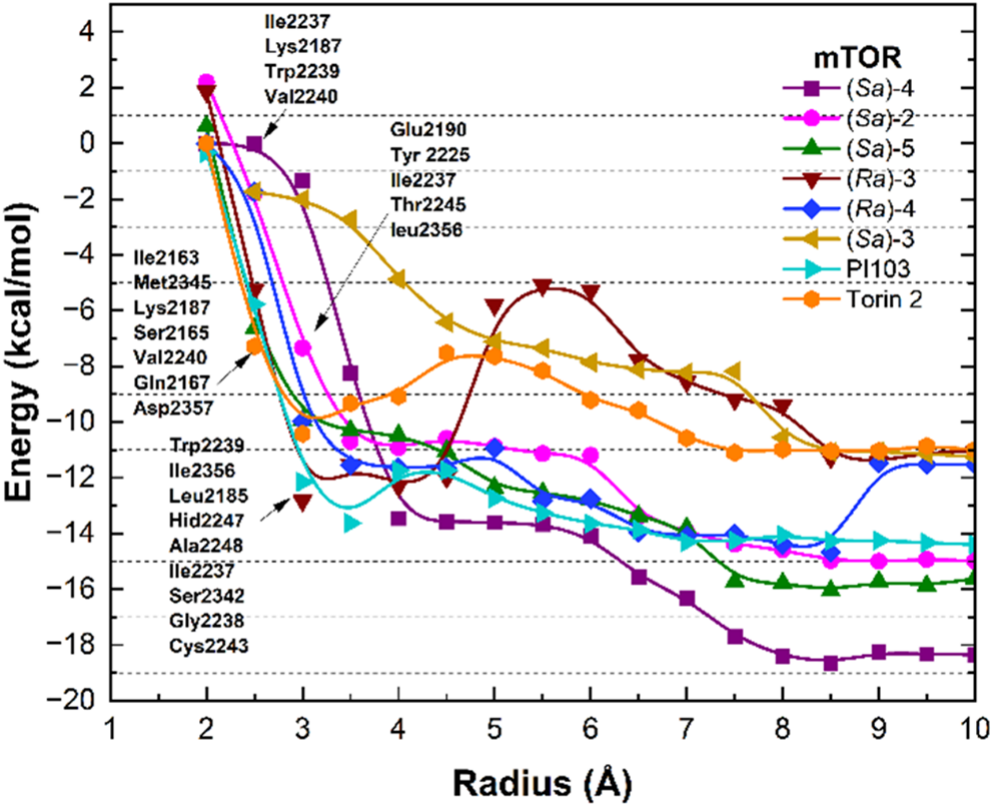
Fluctuation of the total interaction energy in mTOR complexes. Interaction energy is expressed in kcal/mol and distance in angstroms.

As shown in Figure 6, residues Trp2239, Ile2356, Leu2185, Ile2237, Ile2163, Trp2245, and Met2345 seem to be responsible for most of the attractive interactions in mTOR complexes, while residues Asp2357, Glu2190, Lys2187, and Gly2238 interact repulsively with some of the tested configurations. For completeness, the individual amino acid contributions of all residues are listed in Tables S10–S15 (in the Supporting Information).

**Figure 6:**
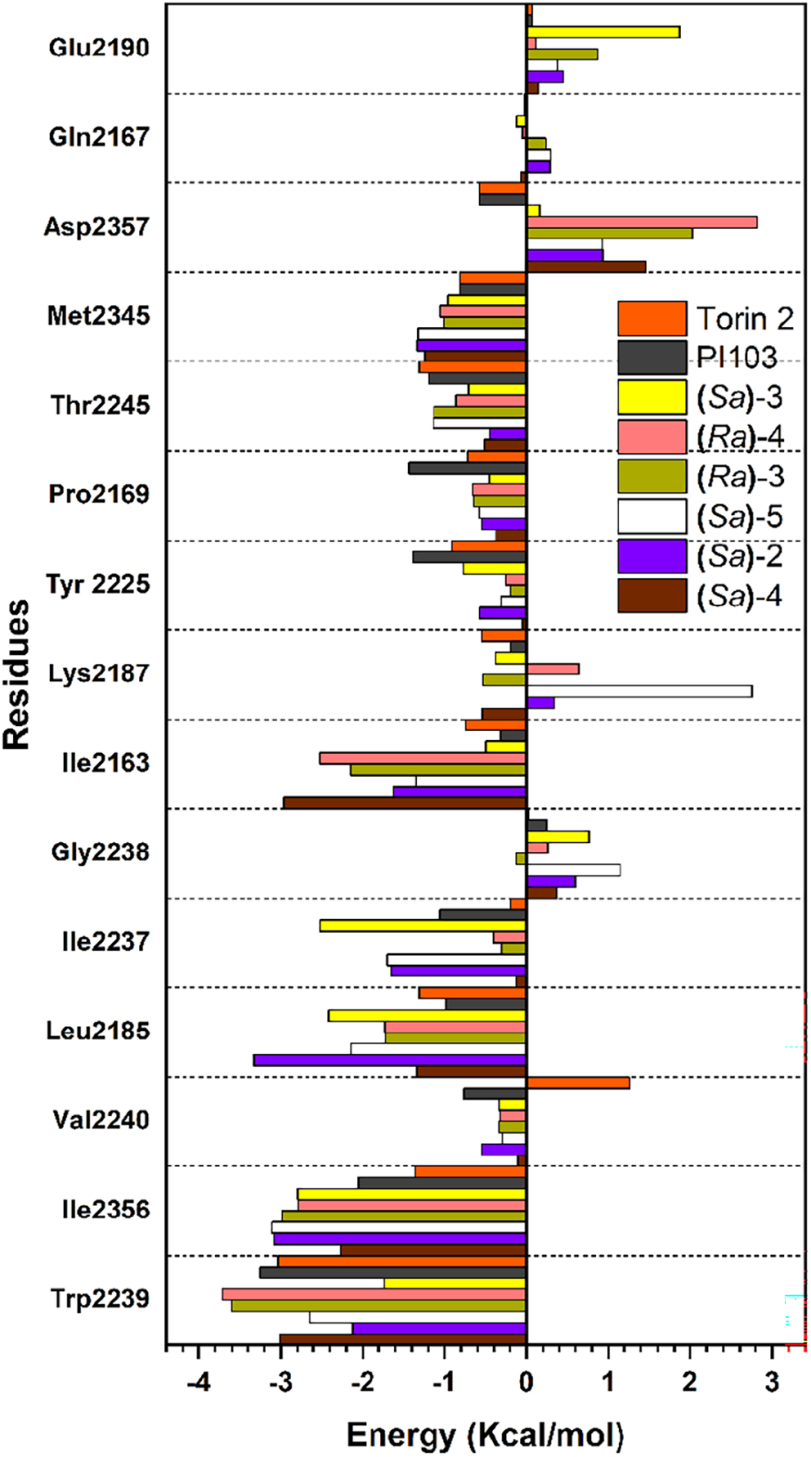
Individual interaction energy of residues at the ATP-binding site of mTOR.

A visual inspection is available in Figure 7 (A–F), which shows that configuration (*Sa*)-**2** forms strong hydrophobic interactions with Ile2163, Ile2237, and Ile2356 through its aromatic rings in regions i and vi. Met2345 forms a π–sulfur interaction with the aromatic ring in region i, while Trp2239 forms a T-shaped π–π interaction with the aromatic ring in region ii. In region iv, both the hydroxyl and the ketone group form hydrogen bonds with Ser2165, while the oxygen atom in the methoxy group of region i forms a hydrogen bond with the amide nitrogen in the peptide bond between Ile2356 and Asp2357. Configuration (*Ra*)-**3** has region i stabilized by two hydrogen bonds formed by the hydroxyl group with Asp2357 and Lys2187. Region ii is stabilized by two hydrogen bonds formed by the hydroxyl and ketone groups with the carbonyl group (C=O) and the amide nitrogen of Val2240. In addition, one face of the aromatic ring is stabilized by a π–π interaction with Trp2239 and the other face is stabilized by a π–sulfur interaction with Met2345. Region iv is stabilized by a hydrogen bond with His2340. Interestingly, in the (*Sa*)-**3** configuration, the binding orientation of this compound flipped, so that, in region i, the hydroxyl group formed a hydrogen bond with the amide nitrogen of Val2240, and the aromatic ring was stabilized by a T-shaped π–π interaction with Trp2239 and a π–sulfur interaction with Met2345. Region ii is stabilized by a hydrogen bond between the keto group and the amide nitrogen of Asp2357. Configuration (*Ra*)-**4** binds with a geometry similar to (*Ra*)-**3**, with the oxygen atom of the methoxy group in region i forming two hydrogen bonds, one with Lys2187 and another with Asp2357. In region ii, the noreugenin moiety is stabilized by a π–π interaction with Trp2239 and the other face by a π–sulfur interaction with Met2345, in addition to two hydrogen bonds formed between the hydroxyl and ketone groups with the carbonyl group (C=O) and the amide nitrogen of Val2240, respectively. Region iv is stabilized by a hydrogen bond between the keto group and Ser2342. Region i of configuration (*Sa*)-**4** is stabilized by an interaction between the methoxy group and Asp2244, in addition to a T-shaped π–π interaction with Trp2239. Region ii forms a hydrogen bond between the keto group and the amide nitrogen of Val2240, a T-shaped π–π interaction with Trp2239, and a π–sulfur interaction with Met2345. The methoxy group in region iv forms a hydrogen bond with His2340. In the configuration (*Sa*)-**5**, the aromatic ring in region i forms a π–sulfur interaction with Met2345 and a hydrogen bond with the amide nitrogen atom of Val2240. Region ii is stabilized by hydrophobic interactions between the noreugenin rings and residues Ile2356 and Ile2257. In addition, the hydroxyl group forms a hydrogen bond with the amid nitrogen of Asp2357. Region iv forms a T-shaped π–π interaction with Tyr2225 and a π–sulfur interaction with Met234, located on the other side of the cleft.

**Figure 7:**
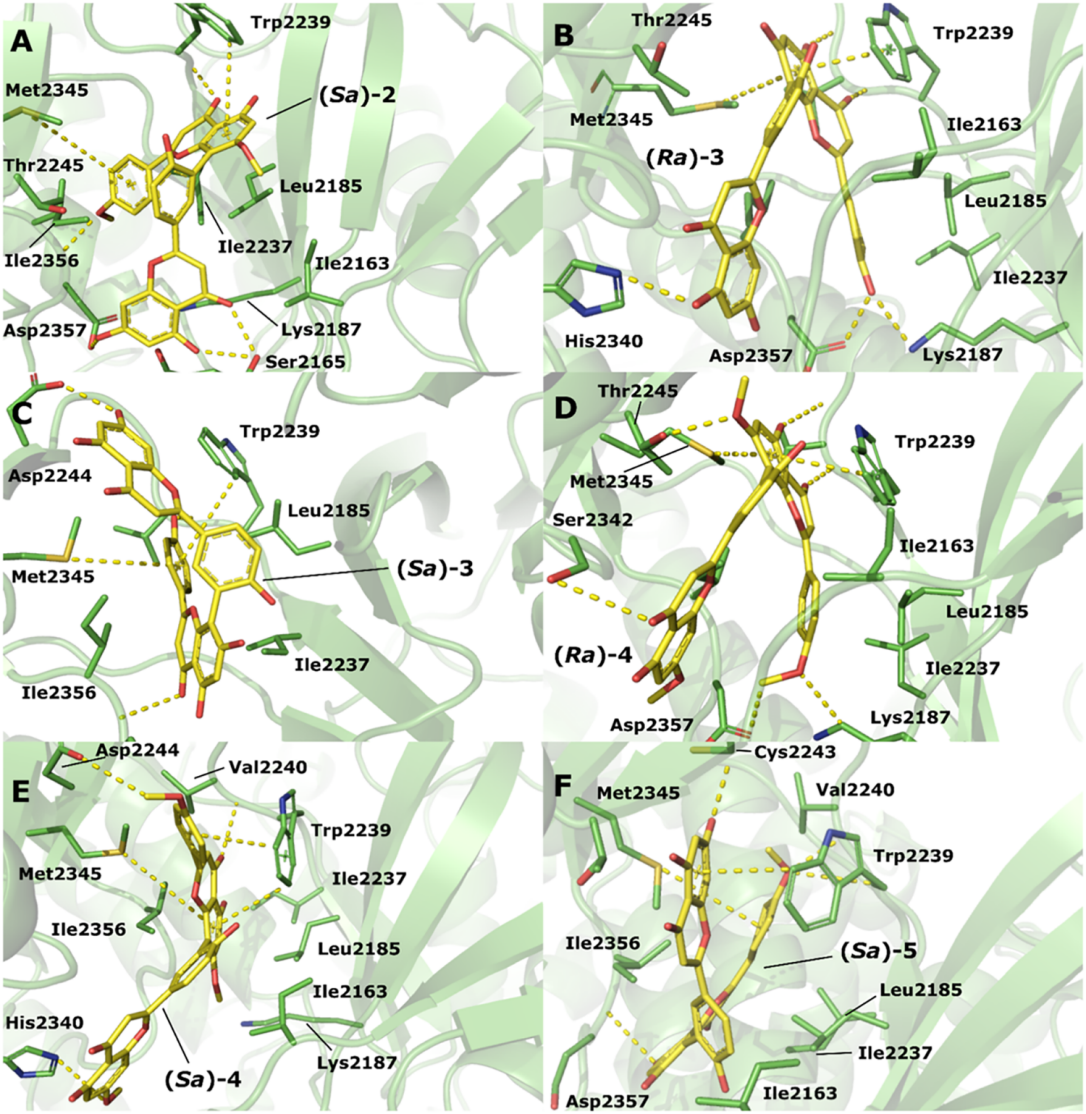
Graphical representation of compounds 2–5 bond to the ATP-binding site of mTOR, showing (A) (*Sa*)-**2** interactions; (B) (*Ra*)-**3** interactions; (C) (*Sa*)-**3** interactions; (D) (*Ra*)-**4** interactions; (E) (*Sa*)-**4** interactions; and (F) (*Sa*)-**5** interactions. Protein structures are illustrated as cartoons, while the residue side chains and ligands are represented as sticks.

The ranking for the calculated binding affinity between compounds 2–5 and PI3Kα is (*Ra*)-**3** > (*Sa*)-**4** > (*Ra*)-**4** > (*Ra*)-**5** > (*Ra*)-**2** > (*Sa*)-**2**, while for mTOR it is (*Sa*)-**5** > (*Sa*)-**2** > (*Sa*)-**4** > (*Ra*)-**4** > (*Sa*)-**3** > (*Ra*)-**3**. Interestingly, results indicated that only (*Sa*)-**2**, (*Ra*)-**3**, (*Sa*)-**4**, and (*Ra*)-**4** behaved as dual inhibitors, highlighting that the sense of chirality (*Ra* or *Sa*) in bioflavonoid molecules plays a crucial role in enzyme inhibition, opening promising new possibilities for the rational design of PI3Kα/mTOR dual inhibitors.

Altogether, this study highlights the effectiveness of a multiapproach silico strategy combining ensemble-based virtual screening, ADME profiling, molecular dynamics simulations, and MM/PBSA free-energy calculations for the identification and prioritization of biflavonoid scaffolds targeting the active sites of both proteins, PI3Kα and mTOR. Although these computational findings provide valuable insights into binding potential, selectivity, and drug-likeness, we acknowledge that experimental validation is essential to confirm biological activity, metabolic stability, and pharmacokinetic behavior. Future studies should focus on synthesizing the prioritized biflavonoids and evaluating their efficacy through *in vitro* kinase assays and selectivity profiling. Nonetheless, the results presented here offer a robust and cost-effective platform for early-stage drug discovery and support the continued investigation of biflavonoids as dual inhibitors of PI3K and mTOR.

## 3 Conclusions

In this study, we identified four bioflavonoids with dual inhibitory potential for PI3Kα/mTOR, derived from a virtual screening of 1745 phytochemicals from the Brazilian NuBBE database. All selected compounds exhibited enantioselective binding, highlighting the influence of their atropisomeric configurations on the binding affinity. Among them, compound 4 stood out for its dual inhibitory activity in both configurations ((*Sa*) and (*Ra*)). In addition, configurations (*Sa*)-**2**, (*Ra*)-**3**, (*Sa*)-**4**, and (*Ra*)-**4** also behaved as dual inhibitors, showing meaningful binding to both PI3Kα and mTOR. On the other hand, (*Sa*)-**3** and (*Sa*)-**5** showed a preference for mTOR, while (*Ra*)-**2** and (*Ra*)-**5** exhibited selectivity for PI3Kα.

Binding energy analyses and residue-level interaction mapping revealed that dual inhibition was supported by key stabilizing interactions within 2.5 to 4.5 Å of the active sites. For PI3Kα, hydrophobic contacts with Ile800, Ile848, and Ile932, π–π stacking with Trp780, and π–sulfur interactions with Met772 and Met922 were particularly important, while Asp933 consistently contributed to repulsive interactions. In mTOR, stabilizing contacts were primarily mediated by Trp2239, Ile2356, Leu2185, Ile2237, and Ile2163, whereas repulsive interactions involving Asp2357, Glu2190, Lys2187, and Gly2238 varied by configuration. These findings reveal distinct binding modes and highlight the structural basis for selectivity and dual-target potential.

Altogether, these findings underscore the importance of chirality in modulating enzyme inhibition and provide a solid foundation for the rational design of dual PI3Kα/mTOR inhibitors. Furthermore, this study supports the potential of plant-derived natural products as promising leads for anticancer drug development. Experimental validation, including selectivity profiling, SAR analysis, and pharmacokinetic studies, will be crucial in the next stages.

## 4 Computational Details

Ensemble-based virtual screening targeting human PI3Kα and mTOR was performed by using a conformational set based on crystallographic geometries of both enzymes. For the sake of completeness, states representing both enzymes in the bound and unbound (APO) states were employed during the screening. A total of 1745 natural compounds of plant origin were docked against each enzyme’s ensemble. Among the compounds with a dual inhibitory profile, 11 showed significantly high binding energy. Of these, four exhibited atropisomerism and underwent stereoselectivity tests. Subsequently, the resulting binding geometries were subjected to molecular dynamics simulations to enhance the binding interactions. For this purpose, energy decomposition calculations were performed.

### 4.1 Structural Data

The following crystallographic structures were retrieved from the Protein Data Bank (PDB): 4JPS,^34^ 4L23,^12^ 4L2Y,^12^ 4YKN,^35^ 4ZOP (Knapp et al., 2015), 5DXH,^36^ 5DXT,^36^ 5XGH,^37^ 5XGI (Song et al., 2018), 3JBZ,^38^ 4JT5,^39^ 4JT6, 4JSX, and 4JSV.^39^ Coordinates of small plant-derived compounds were obtained from the Brazilian database NuBBE.

### 4.2 Protein and Ligand Preparation

Prior to simulations, all protein structures were aligned by α carbons and protonated at pH 7.0 using the ProPKA code on the PDB 2PQR web server.^40^ Small compounds were protonated at pH 7.0 using Open Babel v.2.3.1 software.^41^ When necessary, structures of axial isomers were prepared using the Chemaxon package and energy minimized using Avogadro.^42^ Structures were converted to the PDBQT format using the AutodockTools algorithm.

### 4.3 Ensemble Generation

For system validation, self-docking and cross-docking techniques were used. The results of self-docking and cross-docking were considered satisfactory when the lowest energy poses had a root mean square below 3Å when compared to the position of the reference structure.

Self-docking consists of making the molecular docking between a protein with an experimentally resolved structure and the ligand crystallized along with it. For the study, nine structures of PI3Kα enzyme obtained from the PDB (PDB codes: 4JPS, 4L23, 4L2Y, 4YKN, 4ZOP, 5DXH, 5DXT, 5XGH, 5XGI) and six structures corresponding to the mTOR enzyme were selected (PDB codes: 3JBZ, 4JSV, 4JSX, 4JT5, 4JT6, 4JSP). In the first step in self-docking, the proteins were separated from the ligand using PyMOL v.1.7.5.0 software. In the second step, the proteins were protonated at pH 7.0 by using ProPKA on the PDB 2PQR web server. The ligands were also protonated at pH 7.0 using the Avogadro software.

The molecular docking was performed on a search space (box) comprising the active site of the protein. In this space, the software tries to dock the ligand in a position and conformation that results in the lowest binding energy between the receptor and ligand. The AutodockTools algorithm was used for the preparation of molecular docking. A box was generated; the box sizes 20 × 20 × 20 (*x*, *y*, *z*) point to the center of the mTOR binding site: center_*x* = −17.728, center_*y* = −32.917, center_*z* = −57.784, and exhaustiveness 4. In the PI3Kα protein, the Box was constructed with its center at −1,166, −8,951, and 18,068 (*x*, *y*, *z*). The docking was repeated 10 times for each receptor. The conformations found were compared to the original conformation of the ligand in its crystallographic structure by calculating RMSD.

The cross-docking analysis was important to evaluate a possible influence of the different conformations of the enzymes on the proposed binding mode of molecular docking for the tested inhibitors and to propose sets of enzyme structures. The cross-docking experiment was performed with 9 inhibitors of PI3Kα and 6 inhibitors for mTOR, using the representative structures.

### 4.4 Ensemble-Based Virtual Screening

For the molecular docking study, a total of 1745 compounds were selected. The three-dimensional structures of these inhibitors were taken from the NuBBE database in mol2 format. The compounds were converted into the PDBQT format and protonated using the automation of the Open Babel program.^41^ The docking simulations were repeated ten times for each compound tested.

The molecular docking experiment was performed with the aid of the AutoDock Vina program.^43^ The search area was defined based on the central coordinates of the catalytic sites of the PI3Kα/mTOR enzymes, employing an experimental ligand as a guide. The AutodockTools algorithm was used for the preparation of molecular docking. The same parameters used in self-docking and cross-docking were used for docking with the plant compounds.

Of the 1745 compounds, 10% (174) with the lowest Δ*G* value (kcal/mol) were selected, and from these, only those with a dual inhibition profile (102) were chosen (Supporting Table S1). Next, the best energies were analyzed, and pharmacokinetic analyses were performed using the SwissADME tool from the Swiss Institute of Bioinformatics.^44^ Finally, the 11 best compounds with dual profiles were chosen using the criteria cited above and were subjected to rescoring analyses with MM-PBSA calculations.

### 4.5 Induced Fitting Simulation

Molecular dynamics simulations were used to evaluate the stability of the selected compounds. Molecular dynamics simulations were performed with the aid of the GROMACS 2019 package.^45^ In all systems, the TIP3P water model^46^ was employed to describe water molecules, and the net charge was neutralized by adding Na+ and Cl-ions at 0.15 M concentration.

Prior to the DM production step, total energy minimization was performed by combining the steepest algorithm and the conjugate gradient method in sequence. In total, each system was balanced using integration steps from 1 fs to 2 ns, following gradually decreasing constraint forces over 4 steps of 250 ps each. The long-range electrostatic interaction calculation was modeled with the Particle Mesh Ewald (PME) method^47^ for the temperature coupling, the Nose-Hoover thermostat method was used at 310.15 K, and Parrinello–Rahman barostat^48^ with a reference pressure of 1 atm. The LINCS algorithm was used to constrain the covalent bonds to their equilibrium length.^49^

For the complexes (protein–ligand) of the conformational assembly with compounds of plant origin, a 10 ns trajectory was obtained, which was subsequently used to calculate the interaction energy of the complex (receptor–ligand) system, calculated using the MM-PBSA method. Conformations for MM/PBSA calculations were sampled from two distinct points along the simulation time: the first, immediately after the stabilization of all structures (from 90 to 100 ns), and the second at the end of the simulation (from 190 to 200 ns).

### 4.6 MM-PBSA

Molecular Mechanics Poisson–Boltzmann Surface Area (MM-PBSA) is an efficient method used to estimate the binding free energy in protein–ligand complexes throughout a DM simulation.^50^ The (MM-PBSA) calculations were performed using the code g_mmpbsa,^51^ using the MM-PBSA approach (eq 1), as follows

1 $$\Delta G_{\mathrm{bind}}=G_{\mathrm{complex}}-\left(G_{\mathrm{protein}}+G_{\mathrm{ligand}}\right)$$

where Gcomplex is the total free energy of the protein–ligand complex and Gprotein and Gligand are the total free energies of the isolated protein and ligand in solvent, respectively.

The free energy (G) for each individual unit is estimated in eq 2

2 $$G=\left\langle E_{\mathrm{MM}}\right\rangle +\left\langle G_{\mathrm{solvation}}\right\rangle -\left\langle \mathrm{TS}\right\rangle$$

where *E*_MM_ represents the sum of the internal Molecular Mechanics energy of the molecule, *G*_solvation_ is the solvation free energy, *T* is the temperature in units of Kelvin, and *S* represents the conformational entropy of the System.

The internal energy in Molecular Mechanics is given by the limit and nonlimit terms (eq 3)­

3 $$E_{\mathrm{MM}}=E_{\mathrm{bounded}}+E_{\mathrm{nonb}\mathrm{ou}\mathrm{nded}}$$

where for each individual molecular unit, *E*_bonded_ represents the bonding interactions consisting of dihedral angle bonding and improper interactions. The unbound (*E*_nonbonded_) interactions include both electrostatic (*E*_elec_) and van der Waals interactions and are modeled by using Coulomb and Lennard–Jones (LJ) potential functions, respectively.

The free energy of solvation was calculated using eq 4

4 $$G_{\mathrm{solvation}}=G_{\mathrm{polar}}+G_{\mathrm{nonpolar}}$$

where *G*_solvation_ represents the amount of energy spent to transfer a solute from vacuum to solvent and is calculated using an implicit solvent model. *G*_polar_ is the polar solvation energy of a molecule, estimated by solving the Poisson–Boltzmann (PB) equation, and *G*_nonpolar_ is the term for the nonpolar solvation energy that is approximated by a solvent accessible surface area (SASA) term, based on the assumption that it has a linear dependence on the *G*_nonpolar_ term (eq 5)­

5 $$G_{\mathrm{nonpolar}}=\mathrm{\gamma A}+b$$

where γ is a coefficient related to the surface tension of the solvent, *A* is SASA, and *b* is the fitting parameter.

### 4.7 Total Energy Interaction Analysis and Amino Acid Contributions

The fluctuation of the total interaction energy, considering amino acid residues located within 10 Å of the ligand, was analyzed by summing up the individual interaction energies of residues in steps of 0.5 Å. Python scripts “MmPbSaStat.py” and “MmPbSaDecomp.py”^51^ were used to perform per-residue energy decomposition based on the calculated *E*_MM_, *G*_polar_, and *G*_nonpolar_ energy components of MM-PBSA to identify key amino acid contributions to the binding of the distinct compounds at the ATP-binding site of PI3Kα and mTOR.

## Supplementary Material



## Notes

Plots showing results for the self- and cross-docking simulations, RMSD plots from molecular dynamics, a list of the identified top 10% potential dual-inhibitor compounds, and the calculated MM-PBSA energies per residue (PDF)
