# Supplementary material for: In Silico Identification of Chiral Biflavonoids as Dual PI3Kα/mTOR Inhibitors
Source: ACS Omega. 2025 Sep 19;10(38):44344–55. doi: 10.1021/acsomega.5c06196 (PMC12489665; doi:10.1021/acsomega.5c06196)
Supplement: Supplementary file 1 [file ao5c06196_si_001.pdf]

In silico identification of chiral biflavonoids as dual PI3Kα/mTOR inhibitors

Francisca Fernanda Nunes Azevedo [a], Francisca Joseli Freitas de Sousa [a], Jonatas Martins Negreiro [c], Jaqueline Vieira Carletti [b], Maria Conceição Ferreira Oliveira [c], Geancarlo Zanatta [a,d*]

a - Postgraduate Programme in Biochemistry, Department of Biochemistry at Federal University of Ceará, Fortaleza, CE, Brazil.

b - Department of Biochemistry, Federal University of Rio Grande do Sul, RS, Brazil

c - Department of Organic and Inorganic Chemistry, Science Center, Federal University of Ceará, Fortaleza, CE, Brazil.

d - Postgraduate Programme in Cellular and Molecular Biology at Federal University of Rio Grande do Sul, Porto Alegre, RS, Brazil.

* Corresponding Author: G.Z (geancarlo.zanatta@gmail.com)

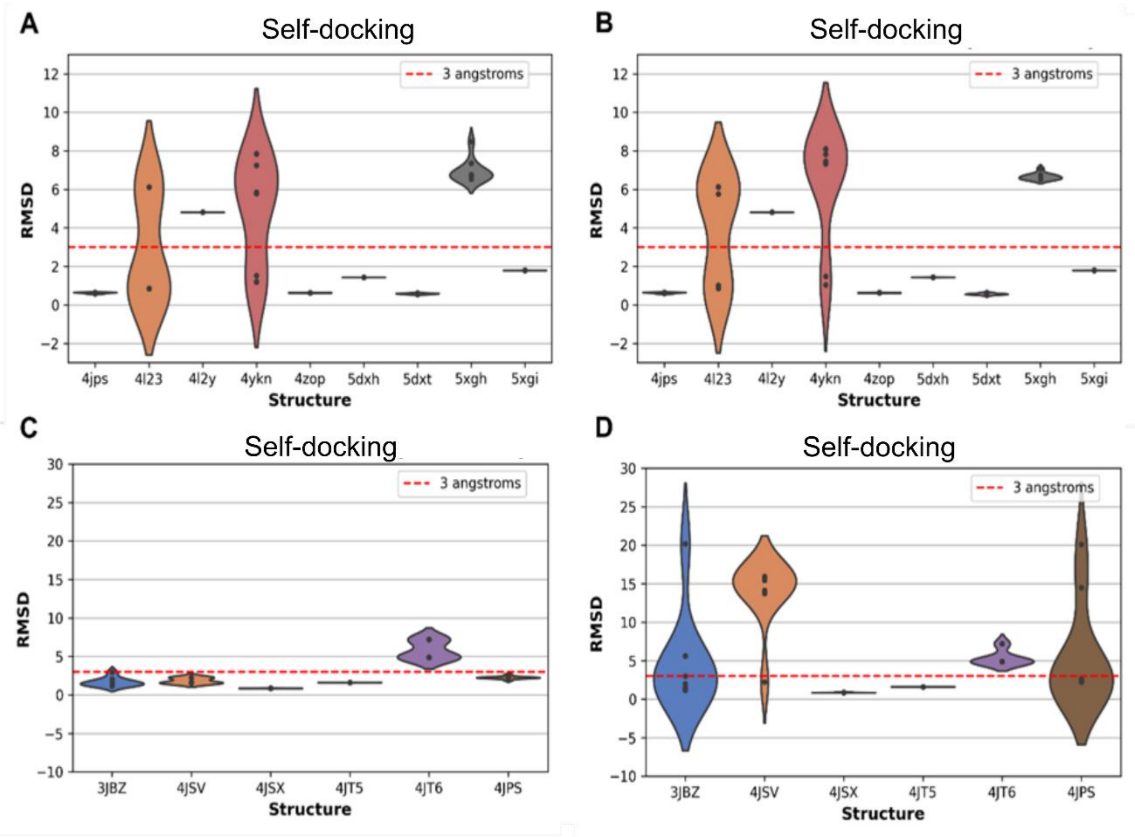

Supplementary figure 1. RMSD distributions for redocking of native ligands into their crystallographic binding sites. Panels (A–B) show PI3Kα results and (C–D) show mTOR results. Each violin plot represents one protein structure (PDB code on x-axis). Docking was performed with a box of 20 × 20 × 20 Å centered at the binding site. The red dashed line marks the 3 Å success threshold. Higher RMSD values for 4L23, 4YKN, and 4JT6 reflect conformational flexibility of the ligand or nearby protein regions.

Supplementary figure 2: Results of crossdocking using PI3Kα crystallographic structures.

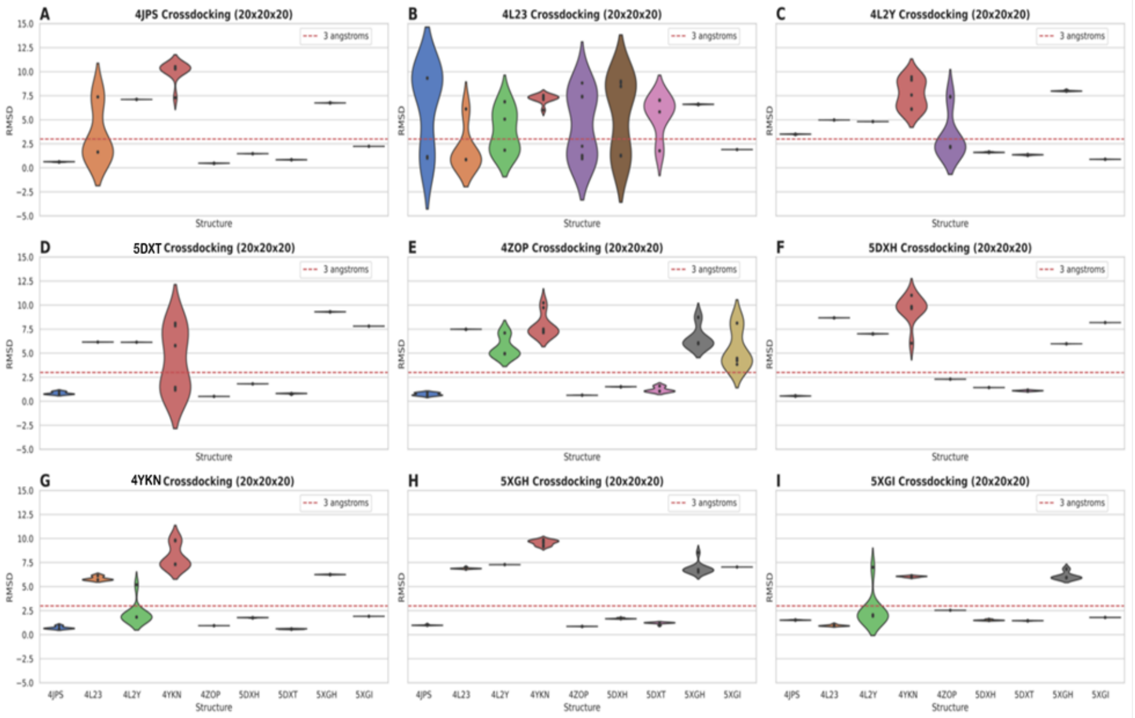

Red line represents the accepted root mean square deviation (RMSD) threshold. Search box was set to dimensions 20x20x20. Violin plots represent values for ten repetitions.

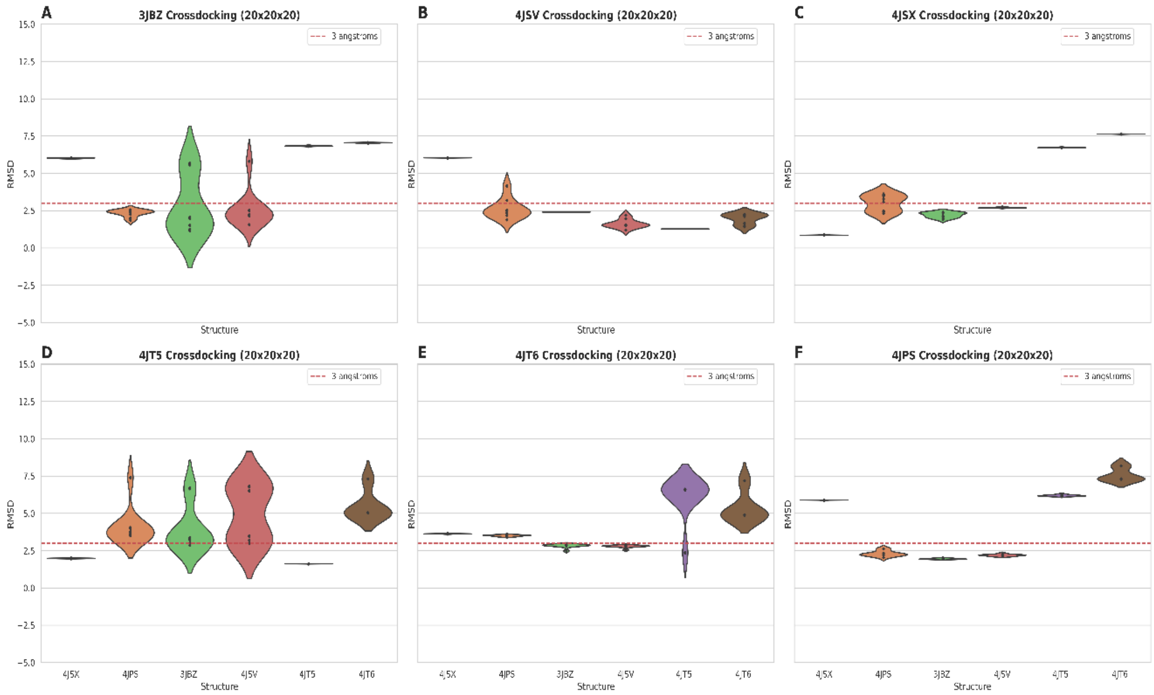

Supplementary figure 3: Results of crossdocking using mTOR crystallographic structures. Red line represents the accepted root mean square deviation (RMSD) threshold. Search box was set to dimensions 20x20x20. Violin plots represent values for ten repetitions.

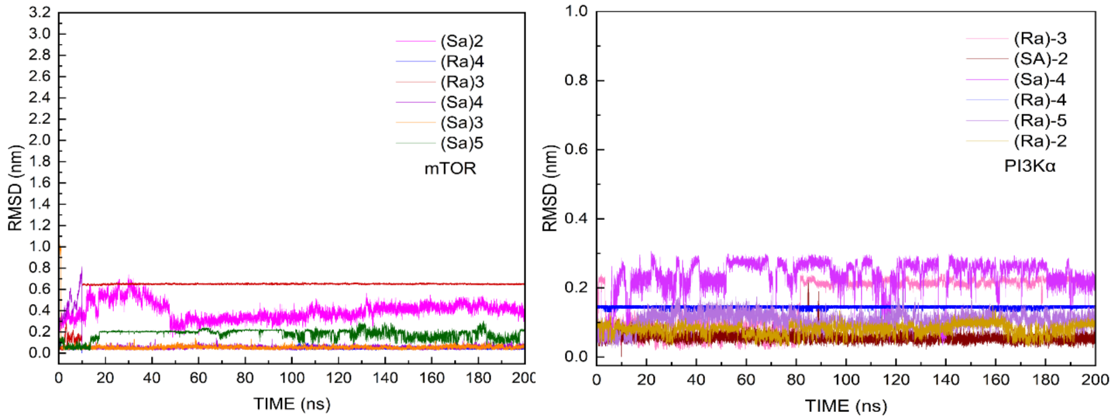

Supplementary figure 4. Evolution of molecular dynamics for mTOR (A) and PI3K (B) complexes along 200-ns trajectories.

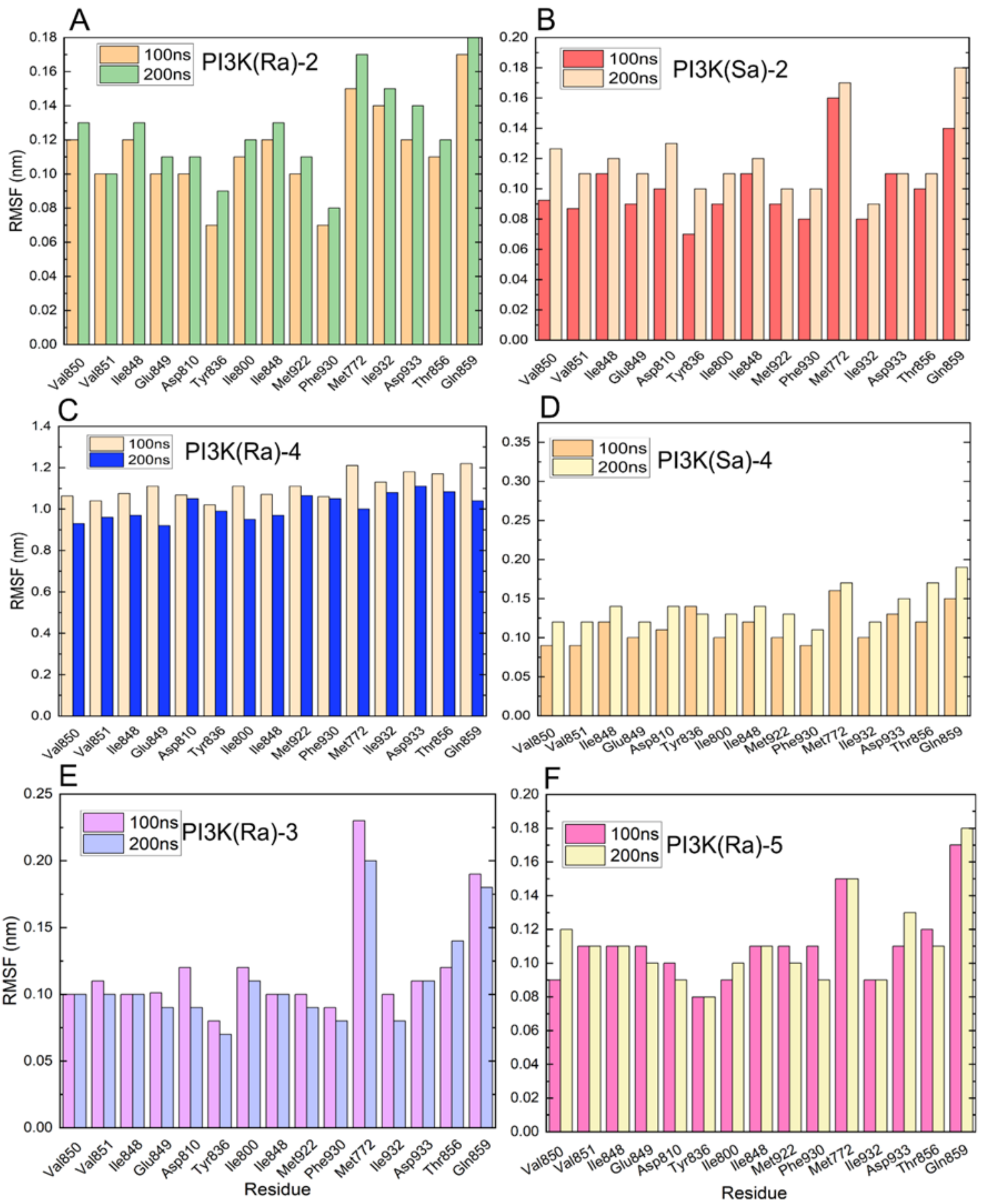

Supplementary figure 5. RMSF of the residues of the ATP binding site of PI3Kα, considering trajectories of 100 and 200 ns

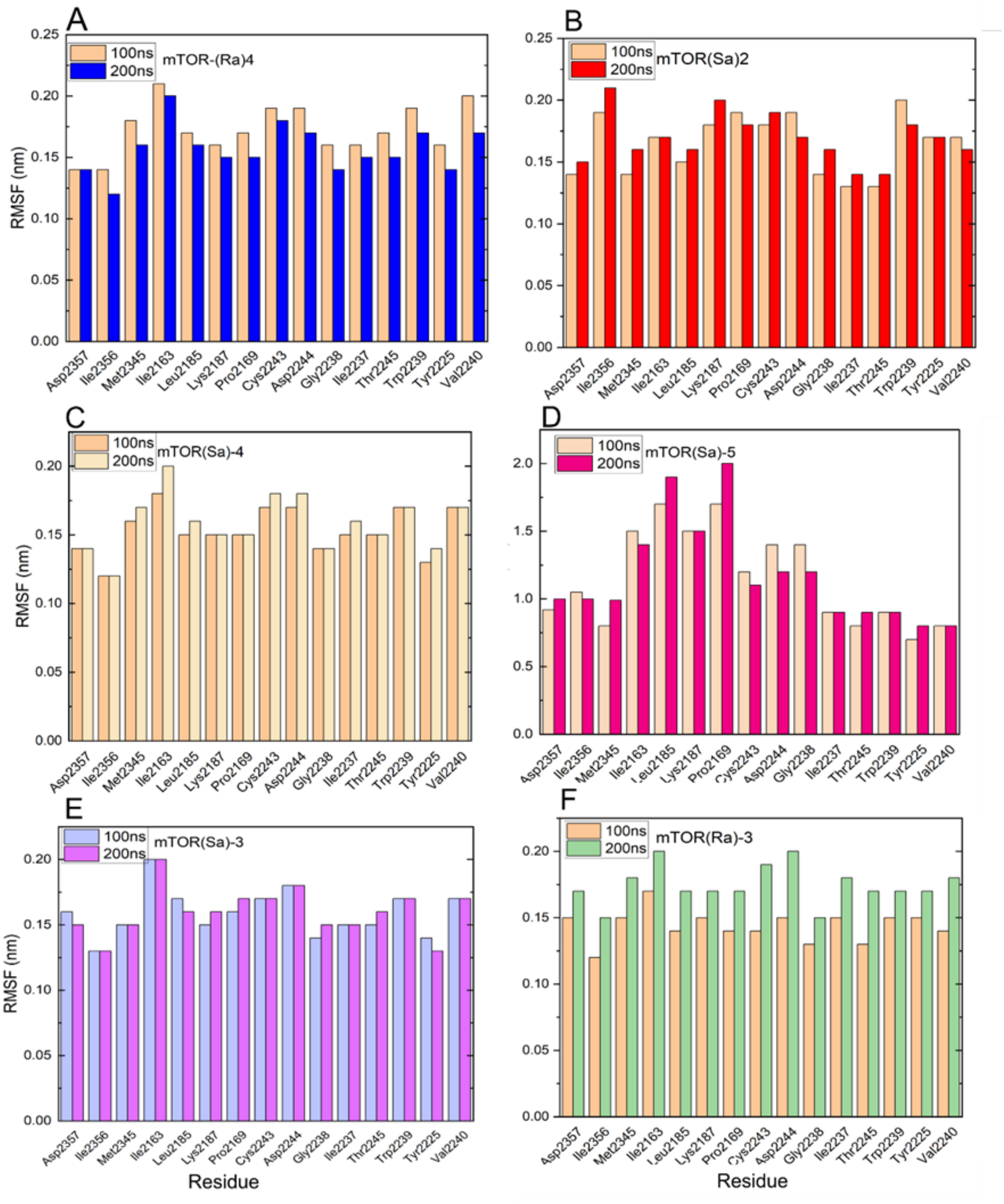

Supplementary figure 6. RMSF of the residues of the ATP binding site of mTOR, considering trajectories of 100 and 200 ns.

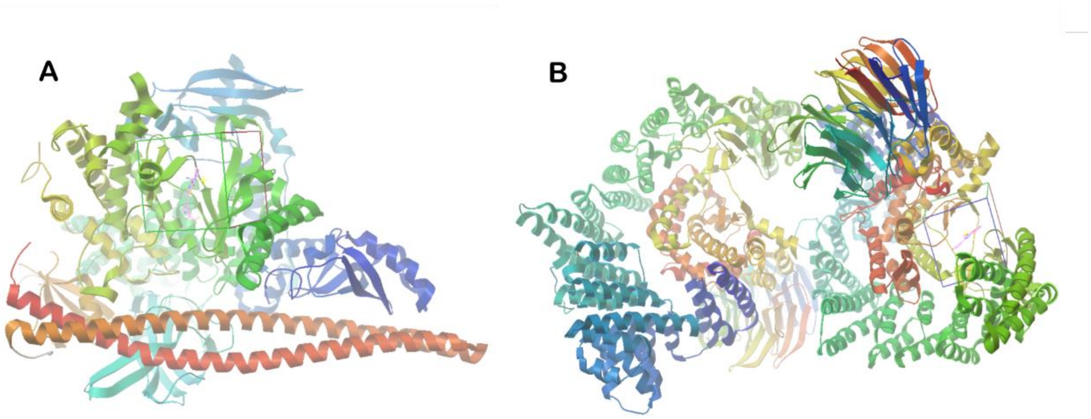

Supplementary figure 7:Representation of the docking box (20 × 20 × 20) used for PI3K and mTOR. This figure illustrates the coordinates and dimensions applied in the docking experiments.

Supplementary table1 – Selected 102 compounds.

NuBBE _ID	**Chemical Class**	**Common Name**	**IUPAC Name**
NuBBE_387	Terpenes Triterpenoid	3β-O-trans-p-coumaroyl-2α-hydroxy-urs-12-en-28-oic acid	(1S,2R,6aS,6bR,10S,11S,12aR)-11-hydroxy-10-{[(2E)-3-(4-hydroxyphenyl)prop-2-enoyl]oxy}-1,2,6a,6b,9,9,12a-heptamethyl-1,2,3,4,4a,5,6,6a,6b,7,8,8a,9,10,11,12,12a,12b,13,14b-icosahydropicene-4a-carboxylic acid
NuBBE_198	Flavonoids Biflavonoid	Amentoflavone 7",4'"-dimethyl ether	8-[5-(5,7-dihydroxy-4-oxo-4H-chromen-2-yl)-2-hydroxyphenyl]-5-hydroxy-7-methoxy-2-(4-methoxyphenyl)-4H-chromen-4-one
NuBBE_200	Flavonoids Biflavonoid	Amentoflavone	8-[5-(5,7-dihydroxy-4-oxo-4H-chromen-2-yl)-2-hydroxyphenyl]-5,7-dihydroxy-2-(4-hydroxyphenyl)-4H-chromen-4-one
NuBBE_197	Flavonoids Biflavonoid	Heveaflavone	5-hydroxy-8-[2-hydroxy-5-(5-hydroxy-7-methoxy-4-oxo-4H-chromen-2-yl)phenyl]-7-methoxy-2-(4-methoxyphenyl)-4H-chromen-4-one
NuBBE_199	Flavonoids Biflavonoid	podocarpusflavone	8-[5-(5,7-dihydroxy-4-oxo-4H-chromen-2-yl)-2-hydroxyphenyl]-5,7-dihydroxy-2-(4-methoxyphenyl)-4H-chromen-4-one
NuBBE_1483	Alkaloids Acridine alkaloid or acridone	atalaphyllinine	6,11-dihydroxy-2,2-dimethyl-12-phenyl-5,10-dihydro-2H-1-oxa-5-azatetraphen-10-one
NuBBE_1321	Flavonoids Flavone	3',4'-methylenedioxy-7,8-(2",2"-dimethylpyrano)-flavone	2-(2H-1,3-benzodioxol-5-yl)-8,8-dimethyl-4H,8H-pyrano[2,3-h]chromen-4-one

NuBBE_2171	Flavonoids Flavanone	Xambioona	(4S)-4-(2,2-dimethyl-2H-chromen-6-yl)-12,12-dimethyl-3,11-dioxatricyclo[8.4.0.$0^{2,7}$]tetradeca-1,7,9,13-tetraen-6-one
NuBBE_1265	Flavone	Erythrisenegalone	(2S)-5-hydroxy-2-(4-hydroxyphenyl)-8,8-dimethyl-6-(3-methylbut-2-en-1-yl)-2H,3H,4H,8H-pyrano[2,3-f]chromen-4-one
NuBBE_1320	Flavone	3-Acetylmundulinol	8,8-dimethyl-2-phenyl-4H,8H-pyrano[2,3-h]chromen-4-one
NuBBE_1263	Flavonoids: Flavone	Limonianin	5-hydroxy-2-(4-hydroxyphenyl)-8,8-dimethyl-4H,8H-pyrano[2,3-h]chromen-4-one
NuBBE_1179	Tannins Hydrolysable Tannin	castalagin	(1R,2S,20S,42S,46S)-7,8,9,12,13,14,25,26,27,30,31,32,35,36,37,46-hexadecahydroxy-3,18,21,41,43-pentaoxanonacyclo[27.13.3.$1^{38,42}$.$0^{2,20}$.$0^{5,10}$.$0^{11,16}$.$0^{23,28}$.$0^{33,45}$.$0^{34,39}$]hexatetraconta-5,7,9,11(16),12,14,23(28),24,26,29(45),30,32,34,36,38-pentadecaene-4,17,22,40,44-pentone
NuBBE_1440	Terpenes: Triterpenoid	5α,6β,8α-trihydroxy-28-norisotoonafolin	(2S,3R,4R,7R,10S,12R,14S,15R,17R,19S,20R)-15-(furan-3-yl)-3,4,20-trihydroxy-2,7,14,20-tetramethyl-6,11,18-trioxahexacyclo[10.8.0.$0^{2,10}$.$0^{3,7}$.$0^{14,19}$.$0^{17,19}$]icosane-5,8-dione
NuBBE_1523	Terpenes: Triterpenoid	melianone	(2R,11S,14S,15S)-14-[(3S)-5-(3,3-dimethyloxiran-2-yl)-2-hydroxyoxolan-3-yl]-2,6,6,11,15-pentamethyltetracyclo[8.7.0.$0^{2,7}$.$0^{11,15}$]heptadec-9-en-5-one
NuBBE1029	Flavonoids Flavanol	Catechin-3-O-(3''-O-trans-cinnamoyl)-α-rhamnopyranoside	2R,3S,4S,5R,6R)-2-{[(2R,3S)-5,7-dihydroxy-2-(4-hydroxyphenyl)-3,4-dihydro-2H-1-benzopyran-3-yl]oxy}-3,5-dihydroxy-6-methyloxan-4-yl(2E)-3-phenylprop-2-enoate
NuBBE1035	Terpenes Triterpenoid	3-Oxoursolic acid	(1S,2R,4aS,6aS,6bR,8aR,12aR,12bR,14bS)-1,2,6a,6b,9,9,12a-heptamethyl-10-oxo-1,2,3,4,4a,5,6,6a,6b,7,8,8a,9,10,11,12,12a,12b,13,14b-icosahydropicene-4a-carboxylic
NuBBE1057	Terpenes Triterpenoid	1(S*)-acetoxy-7(R*)-hydroxy-7-deoxoinchangin	12',15'-dioxaspiro[oxane-3,6'-tetracyclo[8.5.0.$0^{1,14}$.$0^{2,7}$]pentadecane]-4-yl acetate
NuBBE1061	Terpenes Triterpenoid	α-amyrenonol	4aR,6aS,6bR,10S,12aS)-10-hydroxy-2,2,4a,6a,6b,9,9,12a-octamethyl-1,2,3,4,4a,5,6,6a,6b,7,8,8a,9,10,11,12,12a,12b,13,14b-icosahydropicen-13-one
NuBBE1062	Terpenes Triterpenoid	β-amyrenonol	4aR,6aS,6bR,10S,12aS)-10-hydroxy-2,2,4a,6a,6b,9,9,12a-octamethyl-1,2,3,4,4a,5,6,6a,6b,7,8,8a,9,10,11,12,12a,12b,13,14b-icosahydropicen-13-one

NuBBE1063	Terpenes Triterpenoid	3α-hydroxy-ursan-12-one	3S,6aR,6bR,8aR,11R,12S,14bR)-3-hydroxy-4,4,6a,6b,8a,11,12,14b-octamethyl-docosahydropicen-13-one
NuBBE1064	Terpenes : Triterpenoid	3α-hydroxy-12,13-epoxy-oleanane	(1R,3S,6R,9S,14R,15S,18R)-6,10,10,14,15,18,21,21-octamethyl-2-oxahexacyclo[13.8.0.0^{1,3}.0^{5,14}.0^{6,11}.0^{18,23}]tricosan-9-ol
NuBBE1072	Terpenes	Triterpenoid	(1R,2R,5R,8R,14R,17S)-8-(2-hydroxypropan-2-yl)-1,2,5,14,18,18-hexamethylpentacyclo[11.8.0.0^{2,10}.0^{5,9}.0^{14,19}]henicosan-17-ol
NuBBE1095	Terpenes Triterpenoid	Gedunin	(1S,2R,4S,7R,8S,11R,12R,17R,19R)-7-(furan-3-yl)-1,8,12,16,16-pentamethyl-5,15-dioxo-3,6-dioxapentacyclo[9.8.0.0^{2,4}.0^{2,8}.0^{12,17}]nonadec-13-en-19-yl acetate
NuBBE1096	Terpenes Triterpenoid	7-deacetylgedunin; 7-deacetoxygedunin	1S,2R,4S,7R,8S,11R,12R,17R,19R)-7-(furan-3-yl)-19-hydroxy-1,8,12,16,16-pentamethyl-3,6-dioxapentacyclo[9.8.0.0^{2,4}.0^{2,8}.0^{12,17}]nonadec-13-ene-5,15-dione
NuBBE1105	Alkaloids Quinazoline alkaloid	3-(2-(7,7-dimethyl-3,7-dihydropyrano[3,2-e]indol-1-yl)ethyl)quinazoline-2,4(1H,3H)-dione	3-(2-{7,7-dimethyl-3H,7H-chromeno[6,5-b]pyrrol-1-yl}ethyl)-1,2,3,4-tetrahydroquinazoline-2,4-dione
NuBBE1106	Alkaloids Quinazoline alkaloid	3-(2-(7,7-dimethyl-3,7-dihydropyrano[3,2-e]indol-1-yl)ethyl)-1-hydroxyquinazoline-2,4(1H,3H)-dione	3-(2-{7,7-dimethyl-3H,7H-chromeno[6,5-b]pyrrol-1-yl}ethyl)-1-hydroxy-1,2,3,4-tetrahydroquinazoline-2,4-dione
NuBBE1107	Alkaloids Quinazoline alkaloid	3-(2-(7,7-dimethyl-3,7-dihydropyrano[3,2-e]indol-1-yl)ethyl-1-methylquinazoline-2,4(1H,3H)-dione	3-(2-{7,7-dimethyl-3H,7H-chromeno[6,5-b]pyrrol-1-yl}ethyl)-1-methyl-1,2,3,4-tetrahydroquinazoline-2,4-dione
NuBBE1110	Terpenes Triterpenoid	Germanicol	(3S,6aS,6bR,8aR,14aR,14bS)-4,4,6a,6b,8a,11,11,14a,14b-nonamethyl-1,2,3,4,4a,5,6,6a,6b,7,8,8a,9,10,11,12b,13,14,14a,14b-icosahydropicen-3-ol
NuBBE1120	Terpenes Triterpenoid	Photogedunin	1S,2R,4S,7R,8S,12R,19R)-7-(5-hydroxy-2-oxo-2,5-dihydrofuran-3-yl)-1,8,12,16,16-pentamethyl-5,15-dioxo-3,6-

			dioxapentacyclo[9.8.0.$0^{2,4}$.$0^{2,8}$.$0^{12,17}$]nonadec-13-en-19-yl acetatel
NuBBE1131	Terpenes Triterpenoid	methylene-26-hydroxycycloartan-3-one	(1R,3R,12S,15R,16R)-15-[(2R)-7-hydroxy-6-methyl-5-methylideneheptan-2-yl]-7,7,12,16-tetramethylpentacyclo[9.7.0.$0^{1,3}$.$0^{3,8}$.$0^{12,16}$]octadecan-6-one
NuBBE1138	Alkaloids : Indole alkaloid	Raputindole A	yl)-1H,5H,6H,7H-cyclopenta[f]indol-5-yl]ethenyl]-1H-indole
NuBBE1139	Alkaloids : Indole alkaloid	Raputindole D	[(5R,7S)-5-[(E)-2-(1H-indol-5-yl)ethenyl]-7-(2-methylprop-1-en-1-yl)-1H,5H,6H,7H-cyclopenta[f]indol-5-yl]methanol
NuBBE1158	Terpenes : Triterpenoid	limonexic acid	(1R,2R,7S,10R,13R,14R,16S,19R,20S)-19-(5-hydroxy-2-oxo-2,5-dihydrofuran-3-yl)-9,9,13,20-tetramethyl-4,8,15,18-tetraoxahexacyclo[11.9.0.$0^{2,7}$.$0^{2,10}$.$0^{14,16}$.$0^{14,20}$]docosane-5,12,17-trione
NuBBE1160	Terpenes : Triterpenoid	Limonin	2R,7S,10R,13R,14R,16S,19R,20S)-19-(furan-3-yl)-9,9,13,20-tetramethyl-4,8,15,18-tetraoxahexacyclo[11.9.0.$0^{2,7}$.$0^{2,10}$.$0^{14,16}$.$0^{14,20}$]docosane-5,12,17-trione
NuBBE1166	Flavonoids Flavanone	Hesperidin	(2S)-5-hydroxy-2-(3-hydroxy-4-methoxyphenyl)-7-{[(2S,3R,4S,5S,6R)-3,4,5-trihydroxy-6-({[(2R,3R,4R,5R,6S)-3,4,5-trihydroxy-6-methyloxan-2-yl]oxy}methyl)oxan-2-yl]oxy}-3,4-dihydro-2H-1-benzopyran-4-one
NuBBE1178	Tannins: Hydrolysable Tannin	casuarinin	10-[(19S)-2,3,4,7,8,9,19-heptahydroxy-12,17-dioxo-13,16-dioxatetracyclo[13.3.1.$0^{5,18}$.$0^{6,11}$]nonadeca-1,3,5(18),6,8,10-hexaen-14-yl]-3,4,5,17,18,19-hexahydroxy-8,14-dioxo-9,13-dioxatricyclo[13.4.0.$0^{2,7}$]nonadeca-1(19),2,4,6,15,17-hexaen-11-yl 3,4,5-trihydroxybenzoate
NuBBE1181	Terpenes : Triterpenoid	21-hydroxycedrelonelide	1R,2S,4R,6R,7S,10S,11R)-17-hydroxy-6-(2-hydroxy-5-oxo-2,5-dihydrofuran-3-yl)-7,11,15,15-tetramethyl-3-oxapentacyclo[8.8.0.$0^{2,4}$.$0^{2,7}$.$0^{11,16}$]octadeca-12,16-diene-14,18-dione
NuBBE1182	Aromatic derivatives	Ellagic acid 4-O-α-L-4''-O-acetylrhamnopyranoside	(2S,3R,4S,5R,6S)-4,5-dihydroxy-2-methyl-6-({7,13,14-trihydroxy-3,10-dioxo-2,9-dioxatetracyclo[6.6.2.$0^{4,16}$.$0^{11,15}$]hexadeca-1(15),4,6,8(16),11,13-hexaen-6-yl}oxy)oxan-3-yl
NuBBE1183	Terpenes Triterpenoid	23-hydroxycedrelonelide	(1R,2S,4R,6R,7S,10S,11R)-17-hydroxy-6-(5-hydroxy-2-oxo-2,5-dihydrofuran-3-yl)-7,11,15,15-tetramethyl-3-oxapentacyclo[8.8.0.$0^{2,4}$.$0^{2,7}$.$0^{11,16}$]octadeca-12,16-diene-14,18-dione
NuBBE1203	Terpenes Triterpenoid	2α,3α-dihydroxyolea	(4aS,6aS,6bR,10S,11R,12aR)-10,11-dihydroxy-2,2,6a,6b,9,9,12a-heptamethyl-

		n-12- en-28-oic acid	1,2,3,4,4a,5,6,6a,6b,7,8,8a,9,10,11,12,12a,12b,13,14b-icosahydropicene-4a-carboxylic acid
NuBBE1208	Chalcones -	1-(5,7-dihydroxy-2,2-dimethylchroman-6-yl)-3-(1,1,4a-trimethyl-2,3,4,4a,9a-hexahydro-1H-xanthen-7-yl)propan-1-one	3-(1,1,4a-trimethyl-2,3,4,4a,9,9a-hexahydro-1H-xanthen-7-yl)-1-(5,7-dihydroxy-2,2-dimethyl-3,4-dihydro-2H-1-benzopyran-6-yl)propan-1-one
NuBBE1242	Aromatic derivatives Aromatic acid	ellagic acid 4-O-α-L-rhamnopyranoside; eschweilenol C	6,7,14-trihydroxy-13-{[(2S,3R,4R,5R,6S)-3,4,5-trihydroxy-6-methyloxan-2-yl]oxy}-2,9-dioxatetracyclo[6.6.2.0⁴,¹⁶.0¹¹,¹⁵]hexadeca-1(15),4,6,8(16),11,13-hexaene-3,10-dione
NuBBE1243	Flavonoids: Flavone	Isoorientin	2-(3,4-dihydroxyphenyl)-5,7-dihydroxy-6-[(2S,3R,4R,5S,6R)-3,4,5-trihydroxy-6-(hydroxymethyl)oxan-2-yl]-4H-chromen-4-one
NuBBE125	Flavonoids Flavone	Nitensoside B; Pedalitin 6-O-α-rhamnopyranosyl(1'''→6'')-β-glucopyranoside	2-(3,4-dihydroxyphenyl)-5,7-dihydroxy-6-[(2S,3R,4R,5S,6R)-3,4,5-trihydroxy-6-(hydroxymethyl)oxan-2-yl]-4H-chromen-4-one
NuBBE1251	Aromatic derivatives	Ellagic acid 4-O-α-L-3''-O-acetylrhamnopyranoside	(2S,3S,4R,5R,6S)-3,5-dihydroxy-2-methyl-6-({7,13,14-trihydroxy-3,10-dioxo-2,9-dioxatetracyclo[6.6.2.0⁴,¹⁶.0¹¹,¹⁵]hexadeca-1(15),4,6,8(16),11,13-hexaen-6-yl}oxy)oxan-4-yl acetate
NuBBE1252	Terpenes Triterpenoid	28-β-D-glucopyranosyl-6β-hydroxymaslinate	(2R,3S,4S,5R,6R)-3,4,5,6-tetrahydroxyoxan-2-yl]methyl (4aS,6aS,6bR,8aR,10R,11R,12aR,12bR,14bS)-10,11-dihydroxy-2,2,6a,6b,9,9,12a-heptamethyl-1,2,3,4,4a,5,6,6a,6b,7,8,8a,9,10,11,12,12a,12b,13,14b-icosahydropicene-4a-carboperoxoate
NuBBE1271	Terpenes : Triterpenoid	Limonyl acetate	(1R,2R,7S,10R,13S,14R,16S,19R,20S)-19-(furan-3-yl)-9,9,13,20-tetramethyl-5,17-dioxo-4,8,15,18-tetraoxahexacyclo[11.9.0.0²,⁷.0²,¹⁰.0¹⁴,¹⁶.0¹⁴,²⁰]docosan-12-yl acetate
NuBBE1288	Terpenes Triterpenoid	3-tigloylazadiractol	4,11-dimethyl (1S,4S,5R,6S,7S,8R,11S,12R,14S,15R)-4,7,14-trihydroxy-6-[(1S,2S,6S,8S,9R,11S)-2-hydroxy-11-methyl-5,7,10-trioxatetracyclo[6.3.1.0²,⁶.0⁹,¹¹]dodec-3-en-9-yl]-6-methyl-12-{[(2E)-2-methylbut-2-

			enoyl]oxy}-3,9-dioxatetracyclo[6.6.1.0¹,⁵.0¹¹,¹⁵]pentadecane-4,11-dicarboxylate
NuBBE1334	Terpenes : Triterpenoid	N/A	methyl (2Z)-3-[(1R,2R,4S,7R,8S,10R,12R)-7-(furan-3-yl)-10-hydroxy-1,8,15,15-tetramethyl-5,13,18-trioxo-3,6,14-trioxapentacyclo[9.7.0.0²,⁴.0²,⁸.0¹²,¹⁶]octadecan-12-yl]prop-2-enoate
NuBBE1347	Terpenes Triterpenoid	3β-phenylacetoxyurs-12-ene	(3S,6aR,6bS,8aR,11R,12S,14bR)-4,4,6a,6b,8a,11,12,14b-octamethyl-1,2,3,4,4a,5,6,6a,6b,7,8,8a,9,10,11,12,12a,14,14a,14b-icosahydropicen-3-yl
NuBBE1348	Terpenes Triterpenoid	3β-phenylacetoxyolean- 12-ene	3S,6aR,6bS,8aR,14bR)-4,4,6a,6b,8a,11,11,14b-octamethyl-1,2,3,4,4a,5,6,6a,6b,7,8,8a,9,10,11,12,12a,14,14a,14b-icosahydropicen-3-yl 2-phenylacetate
NuBBE1353	Terpenes Triterpenoid	3α,16β-dihydroxyurs-12-ene	3R,6aR,6bS,8S,8aS,11R,12S,14bR)-4,4,6a,6b,8a,11,12,14b-octamethyl-1,2,3,4,4a,5,6,6a,6b,7,8,8a,9,10,11,12,12a,14,14a,14b-icosahydropicene-3,8-diol
NuBBE1354	Terpenes	Triterpenoid	(3R,6aR,6bS,8S,8aS,14bR)-4,4,6a,6b,8a,11,11,14b-octamethyl-1,2,3,4,4a,5,6,6a,6b,7,8,8a,9,10,11,12,12a,14,14a,14b-icosahydropicene-3,8-diol
NuBBE1431	Terpenes : Triterpenoid	Isomultiflorenol	(3S,6bS,8aR,12bS,14bS)-4,4,6b,8a,11,11,12b,14b-octamethyl-1,2,3,4,4a,5,6,6b,7,8,8a,9,10,11,12,12a,12b,13,14,14b-icosahydropicen-3-ol
NuBBE1442	Terpenes Triterpenoid	toonafolin	(1R,3S,4R,6R,9R,17S)-4-(furan-3-yl)-3,9,14,14,18-pentamethyl-7,10,20-trioxahexacyclo[15.2.1.0³,⁸.0⁶,⁸.0⁹,¹⁹.0¹³,¹⁸]icosane-11,15-dione
NuBBE1456	Aromatic derivatives: Chromane	β-toxicarol	2-(3,4-dihydroxyphenyl)-5,7-dihydroxy-6-[(2S,3R,4R,5S,6R)-3,4,5-trihydroxy-6-(hydroxymethyl)oxan-2-yl]-4H-chromen-4-one
NuBBE1478	Alkaloids Acridine alkaloid or acridone	bis-5-hydroxyacronycine	12-(6,11-dihydroxy-2,2,5-trimethyl-10-oxo-3,4,5,10-tetrahydro-2H-1-oxa-5-azatetraphen-4-yl)-6,11-dihydroxy-2,2,5-trimethyl-5,10-dihydro-2H-1-oxa-5-azatetraphen-10-one
NuBBE1486	Aromatic derivatives: Chromene	ββ-rotenone	
NuBBE1521	Terpenes : Triterpenoid	(21S,23S)-Epoxy-21α-methoxy-7α,24S,25-trihydroxy-	(1S,2R,3R,5R,7S,10S,11R,14R,15S)-15-[(2R,3S,5R)-5-[(1S)-1,2-dihydroxy-2-methylpropyl]-2-methoxyoxolan-3-yl]-3-hydroxy-2,6,6,10-

		4α,4β,8β,10β-tetramethyl-25-dimethyl-14,18-cyclo-5,α13 α,- 14 α,17α-cholestan-3β-N-methylanthranilic acid ester	tetramethylpentacyclo[12.3.1.0^{1,14}.0^{2,11}.0^{5,10}]octadecan-7-yl 2-(methylamino)benzoate
NuBBE1572	Alkaloids Indole alkaloid	Aricine	methyl (1S,15S,16S,20S)-7-methoxy-16-methyl-17-oxa-3,13-diazapentacyclo[11.8.0.0^{2,10}.0^{4,9}.0^{15,20}]henicosa-2(10),4,6,8,18-pentaene-19-carboxylate
NuBBE1602	Terpenes Triterpenoid	bourjotinolone A	(1R,2R,7R,11S,14S,15S)-14-[(3R,5S,6S)-5-hydroxy-6-(2-hydroxypropan-2-yl)oxan-3-yl]-2,6,6,11,15-pentamethyltetracyclo[8.7.0.0^{2,7}.0^{11,15}]heptadec-9-en-5-one
NuBBE1700	Alkaloids	Wilforine	20,22,23,25-tetrakis(acetyloxy)-21-[(acetyloxy)methyl]-26-hydroxy-3,15,26-trimethyl-6,16-dioxo-2,5,17-trioxa-11-azapentacyclo[16.7.1.0^{1,21}.0^{3,24}.0^{7,12}]hexacosa-7(12),8,10-trien-19-yl benzoate
NuBBE1725	Terpenes Triterpenoid	Cabraleahydroxylactone	(5S)-5-[(1R,2R,5S,7R,10R,11R,14S,15R)-5-hydroxy-2,6,6,10,11-pentamethyltetracyclo[8.7.0.0^{2,7}.0^{11,15}]heptadecan-14-yl]-5-methyloxolan-2-one
NuBBE1727	Terpenes Triterpenoid	Cabraleone	2-[(2S,5S)-5-[(1R,2R,5E,7R,10R,11R,14S,15R)-5-[2-(2,4-dinitrophenyl)hydrazin-1-ylidene]-2,6,6,10,11-pentamethyltetracyclo[8.7.0.0^{2,7}.0^{11,15}]heptadecan-14-yl]-5-methyloxolan-2-yl]propan-2-ol
NuBBE1731	Terpenes Triterpenoid	Cabralealactone	(5S)-5-methyl-5-[(1R,2R,7R,10R,11R,14S,15R)-2,6,6,10,11-pentamethyl-5-oxotetracyclo[8.7.0.0^{2,7}.0^{11,15}]heptadecan-14-yl]oxolan-2-one
NuBBE1788	Flavonoids Biflavonoid	Strychnobiflavone	8-[6-(5,7-dihydroxy-3-methoxy-4-oxo-4H-chromen-2-yl)-2,3-dihydroxyphenyl]-2-(3,4-dihydroxyphenyl)-5,7-dihydroxy-3-methoxy-4H-chromen-4-one
NuBBE1826	Terpenes Triterpenoid	Bauerenone	(4aR,6bR,8aR,11R,12S,12aR,12bR,14aR,14bR)-4,4,6b,8a,11,12,12a,12b,14b-nonamethyl-1,2,3,4,4a,5,6b,7,8,8a,9,10,11,12,12a,12b,13,14,14a,14b-icosahydropicen-3-one
NuBBE1827	Terpenes : Triterpenoid	Terebenthifolic acid	1S,2R,4aS,6aS,8aR,12aR,12bR,14aS,14bS)-1,2,6a,9,9,12a,14a-heptamethyl-10-oxo-1,2,3,4,4a,5,6,6a,8,8a,9,10,11,12,12a,12b,13,14,14a,14b-icosahydropicene-4a-carboxylic acid
NuBBE1967	Phenylpropanoids -	Gnetin-E	5-[(2S,3S)-4-hydroxy-6-[(2S,3S)-4-hydroxy-2-(4-hydroxyphenyl)-6-[(E)-2-(4-hydroxyphenyl)ethenyl]-2,3-dihydro-1-

			benzofuran-3-yl]-2-(4-hydroxyphenyl)-2,3-dihydro-1-benzofuran-3-yl]benzene-1,3-diol
NuBBE215	Alkaloids -	N-acetylnonaine	1-{3,5-dioxa-11-azapentacyclo[10.7.1.$0^{2,6}$.$0^{8,20}$.$0^{14,19}$]icosa-1(20),2(6),7,14(19),15,17-hexaen-11-yl}ethan-1-one
NuBBE2229	Chalcones	Rubranine	(2E)-1-{9-hydroxy-1,5,5-trimethyl-6,15-dioxatetracyclo[9.3.1.$0^{4,13}$.$0^{7,12}$]pentadeca-7(12),8,10-trien-8-yl}-3-phenylprop-2-en-1-one
NuBBE2281	Flavonoids: Isoflavonoid	4-O'-prenylalpinum isoflavone	5-hydroxy-8,8-dimethyl-3-{4-[(3-methylbut-2-en-1-yl)oxy]phenyl}-4H,8H-pyrano[3,2-g]chromen-4-one
NuBBE240	Terpenes	α-Amyrin	(3S,4aR,6aR,6bS,8aR,11R,12S,12aR,14aR,14bR)-4,4,6a,6b,8a,11,12,14b-octamethyl-1,2,3,4,4a,5,6,6a,6b,7,8,8a,9,10,11,12,12a,14,14a,14b-icosahydropicen-3-ol
NuBBE2410	Alkaloids Indole alkaloid	Conodurine	methyl (1S,12R,14R,15Z,18R)-12-[(1R,15R,17S,18S)-17-ethyl-6-methoxy-3,13-diazapentacyclo[13.3.1.$0^{2,10}$.$0^{4,9}$.$0^{13,18}$]nonadeca-2(10),4,6,8-tetraen-5-yl]-15-ethylidene-17-methyl-10,17-diazatetracyclo[12.3.1.$0^{3,11}$.$0^{4,9}$]octadeca-3(11),4,6,8-tetraene-18-carboxylate
NuBBE2426	Terpenes Triterpenoid	Friedelan-3α-ol	(3R,4R,4aR,6aS,6bR,8aR,12aR,12bS,14aR,14bS)-4,4a,6b,8a,11,11,12b,14a-octamethyl-docosahydropicen-3-ol
NuBBE2491	Terpenes Triterpenoid	Taraxerone	(4aR,6aR,8aR,12aR,12bS,14aR,14bR)-4,4,6a,8a,11,11,12b,14b-octamethyl-1,2,3,4,4a,5,6,6a,8,8a,9,10,11,12,12a,12b,13,14,14a,14b-icosahydropicen-3-one
NuBBE256	Terpenes Triterpenoid	Maytenin, Tingenone	(6bS,8aS,11R,12aR,12bS,14aR)-3-hydroxy-4,6b,8a,11,12b,14a-hexamethyl-2,6b,7,8,8a,9,10,11,12,12a,12b,13,14,14a-tetradecahydropicene-2,10-dione
NuBBE257	Terpenes : Triterpenoid	20α-Hydroxymaytenin; 20-hydroxy-20-epi-tingenone	1S/C28H36O4/c1-16-17-7-8-20-25(3,18(17)13-19(29)23(16)31)10-12-27(5)21-14-28(6,32)22(30)15-24(21,2)9-11-26(20,27)4/h7-8,13,21,31-32H,9-12,14-15H2,1-6H3/t21-,24+,25+,26-,27+,28+/m1/s1
NuBBE278	Chimarrhoside	Chimarrhoside	5-hydroxy-7-{[5-hydroxy-2-(4-hydroxyphenyl)-4-oxo-3-{[(2S,3R,4S,5R,6R)-3,4,5-trihydroxy-6-({[(2S,3R,4R,5R,6S)-3,4,5-trihydroxy-6-methyloxan-2-yl]oxy}methyl)oxan-2-yl]oxy}-4H-chromen-7-yl]oxy}-2-(4-hydroxyphenyl)-3-{[(2S,3R,4S,5S,6R)-3,4,5-trihydroxy-6-({[(2R,3R,4R,5R,6S)-3,4,5-trihydroxy-6-methyloxan-2-yl]oxy}methyl)oxan-2-yl]oxy}-4H-chromen-4-one

NuBBE286	Flavonoids Flavonol	Kaempferol-3-O-α-L-rhamnopyranosyl-(1-6)-α-L-rhamnopyranosyl-(1-4)-β-D-glucopyranoside	3-{[(2S,3R,4R,5S,6R)-3,4-dihydroxy-6-(hydroxymethyl)-5-{[(2S,3R,4R,5R,6S)-3,4,5-trihydroxy-6-({[(2R,3R,4R,5R,6S)-3,4,5-trihydroxy-6-methyloxan-2-yl]oxy}methyl)oxan-2-yl]oxy}oxan-2-yl]oxy}-5,7-dihydroxy-2-(4-hydroxyphenyl)-4H-chromen-4-one
NuBBE288	Flavonoids Biflavonoid	Procyanidin B-3	(2R,3S,4S)-2-(3,4-dihydroxyphenyl)-4-[(2R,3S)-2-(3,4-dihydroxyphenyl)-3,5,7-trihydroxy-3,4-dihydro-2H-1-benzopyran-8-yl]-3,4-dihydro-2H-1-benzopyran-3,5,7-triol
NuBBE317	Terpenes Triterpenoid	28-O-β-D-glucopyranosyl quinovic acid	1S,2R,4aS,6aR,6bR,8aR,10S,12aR,12bR,14bS)-10-hydroxy-1,2,6b,9,9,12a-hexamethyl-4a-({[(2S,3R,4S,5S,6R)-3,4,5-trihydroxy-6-(hydroxymethyl)oxan-2-yl]oxy}carbonyl)-1,2,3,4,4a,5,6,6a,6b,7,8,8a,9,10,11,12,12a,12b,13,14b-icosahydropicene-6a-carboxylic acid
NuBBE360	Flavonoids Flavonol	Kaempferol 3-O-α-L-rhamnopyranosyl (1→6)-O-[β -D-glucopyranosyl (1→3)-O-α-L-rhamnopyranosyl-(1→2)]-O-β -D-glucopyranosyl	3-{[(2S,3R,4S,5S,6R)-3-{[(2S,3R,4R,5S,6S)-3,5-dihydroxy-6-methyl-4-{[(2S,3R,4S,5S,6R)-3,4,5-trihydroxy-6-({[(2R,3R,4R,5R,6S)-3,4,5-trihydroxy-6-methyloxan-2-yl]oxy}methyl)oxan-2-yl]oxy}oxan-2-yl]oxy}-4,5-dihydroxy-6-(hydroxymethyl)oxan-2-yl]oxy}-5,7-dihydroxy-2-(4-hydroxyphenyl)-4H-chromen-4-one
NuBBE361	Flavonoids Flavonol	Quercetin 3-O-α-L-rhamnopyranosyl (1→6)-O-[β -D-glucopyranosyl (1→3)-O-α-L-rhamnopyranosyl-(1→2)]-O-β -D-glucopyranosyl	3-{[(2S,3R,4S,5S,6R)-3-{[(2S,3R,4R,5S,6S)-3,5-dihydroxy-6-methyl-4-{[(2S,3R,4S,5S,6R)-3,4,5-trihydroxy-6-({[(2R,3R,4R,5R,6S)-3,4,5-trihydroxy-6-methyloxan-2-yl]oxy}methyl)oxan-2-yl]oxy}oxan-2-yl]oxy}-4,5-dihydroxy-6-(hydroxymethyl)oxan-2-yl]oxy}-2-(3,4-dihydroxyphenyl)-5,7-dihydroxy-4H-chromen-4-one
NuBBE364	Terpenes	Triterpenoid	(3S,4aR,6aR,6bS,8R,8aS,12aS,14aR,14bR)-4,4,6a,6b,8a,11,11,14b-octamethyl-1,2,3,4,4a,5,6,6a,6b,7,8,8a,9,10,11,12,12a,14,14a,14b-icosahydropicene-3,8-diol
NuBBE366	Terpenes Triterpenoid	Uvaol	3S,4aR,6aR,6bS,8aS,11R,12S,12aS,14aR,14bR)-8a-(hydroxymethyl)-4,4,6a,6b,11,12,14b-heptamethyl-1,2,3,4,4a,5,6,6a,6b,7,8,8a,9,10,11,12,12a,14,14a,14b-icosahydropicen-3-ol
NuBBE379			

NuBBE381	Terpenes Triterpenoid	22β-Hydroxymaytenin, 22-hydroxytingenone, tingenin B	6bS,8aR,9S,11R,12aS,12bS,14aR)-3,9-dihydroxy-4,6b,8a,11,12b,14a-hexamethyl-2,6b,7,8,8a,9,10,11,12,12a,12b,13,14,14a-tetradecahydropicene-2,10-dione
NuBBE386	Terpenes Triterpenoid	2α,3α-Dihydroxy-urs-12-en-28-oic acid	(1S,2R,6aS,6bR,8aR,10R,11S,12aR,12bR,14bS)-10,11-dihydroxy-1,2,6a,6b,9,9,12a-heptamethyl-1,2,3,4,4a,5,6,6a,6b,7,8,8a,9,10,11,12,12a,12b,13,14b-icosahydropicene-4a-carboxylic acid
NuBBE388	Terpenes Triterpenoid	3β-O-trans-p-coumaroylmaslinic acid	4aS,6aS,6bR,10S,11S,12aR)-11-hydroxy-10-{[(2E)-3-(4-hydroxyphenyl)prop-2-enoyl]oxy}-2,2,6a,6b,9,9,12a-heptamethyl-1,2,3,4,4a,5,6,6a,6b,7,8,8a,9,10,11,12,12a,12b,13,14b-icosahydropicene-4a-carboxylic acid
NuBBE407	Terpenes Triterpenoid	Celastrol	2R,4aS,12bR,14aS,14bR)-10-hydroxy-2,4a,6a,9,12b,14a-hexamethyl-11-oxo-1,2,3,4,4a,5,6,6a,11,12b,13,14,14a,14b-tetradecahydropicene-2-carboxylic acid
NuBBE416	Terpenes Triterpenoid	Celastrol	2R,4aS,12bR,14aS,14bR)-10-hydroxy-2,4a,6a,9,12b,14a-hexamethyl-11-oxo-1,2,3,4,4a,5,6,6a,11,12b,13,14,14a,14b-tetradecahydropicene-2-carboxylic acid
NuBBE437	Terpenes Triterpenoid	3β-O-trans-p-coumaroyl-2α-hydroxy-urs-12-en-28-oic acid	(1S,2R,6aS,6bR,10S,11S,12aR)-11-hydroxy-10-{[(2E)-3-(4-hydroxyphenyl)prop-2-enoyl]oxy}-1,2,6a,6b,9,9,12a-heptamethyl-1,2,3,4,4a,5,6,6a,6b,7,8,8a,9,10,11,12,12a,12b,13,14b-icosahydropicene-4a-carboxylic acid
NuBBE438	Terpenes Triterpenoid	α-Amyrin cinnamate	(4aR,6aR,6bS,8aR,11R,12S,12aR,14aR,14bR)-4,4,6a,6b,8a,11,12,14b-octamethyl-1,2,3,4,4a,5,6,6a,6b,7,8,8a,9,10,11,12,12a,14,14a,14b-icosahydropicen-3-yl (2E)-3-phenylprop-2-enoate
NuBBE54	Aromatic derivatives	Corilagin	(1S,19R,21S,22R,23R)-6,7,8,11,12,13,22,23-octahydroxy-3,16-dioxo-2,17,20-trioxatetracyclo[17.3.1.0^{4,9}.0^{10,15}]tricosa-4,6,8,10(15),11,13-hexaen-21-yl
NuBBE546	Phenylpropanoids	Acteoside; [β-3,4-dihydroxyphenyl)-ethyl]-(3'-O-α-L-rhamnopyranosyl)-(4'-O-cafeoyl)-β-D-glycopyranoside, verbascoside	((2R,3R,4R,5R,6R)-6-[2-(3,4-dihydroxyphenyl)ethoxy]-5-hydroxy-2-(hydroxymethyl)-4-{[(2S,3R,4R,5R,6S)-3,4,5-trihydroxy-6-methyloxan-2-yl]oxy}oxan-3-yl (2E)-3-(3,4-dihydroxyphenyl)prop-2-enoate
NuBBE557	Flavonoids Flavonol	Kaempferol-3-O-α-L-(4''-E-p-coumaroyl)-rhamnoside	(2S,3R,4S,5R,6S)-6-{[5,7-dihydroxy-2-(4-hydroxyphenyl)-4-oxo-4H-chromen-3-yl]oxy}-4,5-dihydroxy-2-methyloxan-3-yl (2E)-3-(4-hydroxyphenyl)prop-2-enoate

NuBBE606	Terpenes Triterpenoid	Lupeol dihydrocinnamate	1R,2R,5R,8R,9R,10R,13R,14R,17S,19R)-1,2,5,14,18,18-hexamethyl-8-(prop-1-en-2-yl)pentacyclo[11.8.0.0^{2,10}.0^{5,9}.0^{14,19}]henicosan-17-yl 3-phenylpropanoate
NuBBE74	Terpenes Triterpenoid	β-amyrenone	(4aR,6aR,6bS,8aR,12aR,14aR,14bR)-4,4,6a,6b,8a,11,11,14b-octamethyl-1,2,3,4,4a,5,6,6a,6b,7,8,8a,9,10,11,12,12a,14,14a,14b-icosahydropicen-3-one
NuBBE75	Terpenes Triterpenoid	Germanicone	(4aR,6aR,6bR,8aR,12bS,14aR,14bR)-4,4,6a,6b,8a,11,11,14b-octamethyl-1,2,3,4,4a,5,6,6a,6b,7,8,8a,9,10,11,12b,13,14,14a,14b-icosahydropicen-3-one
NuBBE87	Terpenes : Triterpenoid	ristimerin; 20α-3-hydroxy-2-oxo-24-nor-friedela-1-10,3,5,7-tetraen-carboxylic acid-29-methylester	methyl (2R,4aS,12bR,14aS,14bR)-10-hydroxy-2,4a,6a,9,12b,14a-hexamethyl-11-oxo-1,2,3,4,4a,5,6,6a,11,12b,13,14,14a,14b-tetradecahydropicene-2-carboxylate

Supplementary table2 – List of the top 10% dual Pi3Kα-mTOR inhibitors from EBVS.

Compound	NuBBE Id	IUPAC name	Class
1	387	(1S,2R,6aS,6bR,10S,11S,12aR)-11-hydroxy-10-{[(2E)-3-(4-hydroxyphenyl)prop-2-enoyl]oxy}-1,2,6a,6b,9,9,12a-heptamethyl-1,2,3,4,4a,5,6,6a,6b,7,8,8a,9,10,11,12,12a,12b,13,14b-icosahydropicene-4a-carboxylic acid	Triterpenoid
2	198	8-[5-(5,7-dihydroxy-4-oxo-4H-chromen-2-yl)-2-hydroxyphenyl]-5-hydroxy-7-methoxy-2-(4-methoxyphenyl)-4H-chromen-4-one	Biflavonoid
3	200	8-[5-(5,7-dihydroxy-4-oxo-4H-chromen-2-yl)-2-hydroxyphenyl]-5,7-dihydroxy-2-(4-hydroxyphenyl)-4H-chromen-4-one	Biflavonoid
4	197	5-hydroxy-8-[2-hydroxy-5-(5-hydroxy-7-methoxy-4-oxo-4H-chromen-2-yl)phenyl]-7-methoxy-2-(4-methoxyphenyl)-4H-chromen-4-one	Biflavonoid
5	199	8-[5-(5,7-dihydroxy-4-oxo-4H-chromen-2-yl)-2-hydroxyphenyl]-5,7-dihydroxy-2-(4-methoxyphenyl)-4H-chromen-4-one	Biflavonoid
6	1483	6,11-dihydroxy-2,2-dimethyl-12-phenyl-5,10-dihydro-2H-1-oxa-5-azatetraphen-10-one	Acridine alkaloid
7	1321	2-(2H-1,3-benzodioxol-5-yl)-8,8-dimethyl-4H,8H-pyrano[2,3-h]chromen-4-one	Flavone
8	2171		

9	1265	(2S)-5-hydroxy-2-(4-hydroxyphenyl)-8,8-dimethyl-6-(3-methylbut-2-en-1-yl)-2H,3H,4H,8H-pyrano[2,3-f]chromen-4-one	Flavone
10	1320	8,8-dimethyl-2-phenyl-4H,8H-pyrano[2,3-h]chromen-4-one	Flavone
11	1263	5-hydroxy-2-(4-hydroxyphenyl)-8,8-dimethyl-4H,8H-pyrano[2,3-h]chromen-4-one	Flavone

Supplementary table 3: Calculated binding free energy of PI3Kα and mTOR. Energies are given in Kcal.mol^{-1}

**PI3Kα**		E_{VW}	$E_{electr.}$	E_{polar}	E_{SAV}	E_{SASA}	ΔG_{bind} (SAV)	ΔG_{bind} (SASA)
Compound (*Sa*)-4	**εprot=2 εsol=80**	-51.28 +/- 0.44	-3.28 +/-0.31	-37.15 +/-0.48	-40.48 +/-0.66	-5.44 +/-0.48	-57.86 +/-0.94	-22.82 +/-0.46
Compound (*Ra*)-4	**εprot=2 εsol=80**	-52.24 +/- 1.15	-2.34 +/- 1.13	-34.40 +/-1.15	-40.18 +/-3.25	-5.55 +/- 0.12	-60.57 +/- 0.8	-25.74 +/- 3.74
Compound (*Sa*)-2	**εprot=2 εsol=80**	-53.70 +/-0.31	-4.59 +/-0.21	-35.56 +/-0.34	-43.06 +/-0.78	-5.64 +/-0.03	-65.72 +/-0.87	-28.38 +/-0.36
Compound (R*a*)-2	**εprot=2 εsol=80**	-55.22 +/- 1.78	-5.32 +/- 0.95	-41.48 +/- 2.12	-55.21 +/- 2.69	-5.93 +/- 0.12	-62.0 +/- 3.14	-24.71 +/- 1.80
Compound (Ra)-3	**εprot=2 εsol=80**	-48.59 +/-0.32	-11.85 +/-0.21	36.98 +/-0.40	-38.84 +/-0.02	-4.81 +/-0.40	-62.33 +/-0.29	-28.28 +/-0.40
Compound 7	**εprot=2 εsol=80**	-42.99 +/-0.24	-4.15 +/-0.12	26.70 +/-0.29	-35.46 +/-0.52	-4.42 +/-0.01	-55.88 +/- 0.66	-24.85 +/-0.37
Compound 8	**εprot=2 εsol=80**	-37.32 +/- 1.22	-2.35 +/- 0.61	22.22 +/- 1.46	-32.25 +/- 2.43	-4.37 +/- 0.11	-49.71 +/- 2.95	-21.81 +/- 1.19
Compound (Ra)-5	**εprot=2 εsol=80**	-48.39 +/-1.61	-14.13 +/- 1.44	36.68 +/- 2.50	35.83 +/- 2.56	-4.78 +/- 0.14	61.68 +/- 2.80	30.66 +/- 1.57
Compound 6	**εprot=2 εsol=80**	-35.17 +/- 1.18	-8.31 +/- 0.92	-24.39 +/- 1.11	-28.60 +/- 2.36	-3.97 +/- 0.08	-47.67 +/- 2.77	-23.06 +/- 1.24
Compound 1	**εprot=2 εsol=80**	-49.69 +/- 0.91	-17.45 +/- 1.84	24.05 +/- 1.21	-36.35 +/- 2.76	-5.20 +/- 0.10	-70.13 +/- 3.23	-38.98 +/- 1.70
Compound 11	**εprot=2 εsol=80**	-39.27 +/- 1.17	-2.17 +/- 1.18	24.05 +/- 1.21	-32.39 +/- 2.11	-4.32 +/- 0.09	-49.78 +/- - 74.12 +/- 4.20 2.42	-21.70 +/- 1.20
Compound 9	**εprot=2 εsol=80**	-39.18 +/- 1.47	-5.23 +/- 1.23	29.84 +/- 2.02	-32.87 +/- 2.24	-4.58 +/- 0.11	-47.44 +/- 2.72	-19.17 +/- 1.38
Compound 10	**εprot=2 εsol=80**	-31.17 +/- 1.07	0.02 +/- 0.58	17.89 +/- 1.54	-25.32 +/- 2.22	-3.93 +/- 0.09	-38.57 +/- 2.45	-17.18 +/- 1.1
**mTOR**		E_{VW}	$E_{electr.}$	E_{polar}	E_{SAV}	E_{SASA}	ΔG_{bind} (SAV)	ΔG_{bind} (SASA)
Compound (*Ra*)-4	**εprot=2 εsol=80**	-63.89 +/- 1.83	-8.79 +/- 0.88	42.98 +/- 1.82	-44.40 +/- 3.64	-5.98 +/- 0.11	-74.12 +/- 4.20	-35.69 +/- 1.42

Compound (S*a*)-4	**εprot=2 εsol=80**	-55.83 +/- 1.62	-6.43 +/- 1.90	36.97 +/- 2.14	-39.51 +/- 3.82	-5.86 +/- 0.12	-64.78 +/- 4.26	-31.13 +/- 2.09
Compound (*Sa*)-2	**εprot=2 εsol=80**	-57.48 +/- 1.46	-12.15 +/- 2.19	43.05 +/- 2.92	-44.62 +/- 2.78	-5.71 +/- 0.11	-71.24 +/- 3.38	-32.32 +/- 1.68
Compound (R*a*)-3	**εprot=2 εsol=80**	-57.44 +/- 1.33	-13.25 +/- 1.22	-46.40 +/- 0.10	-44.22 +/- 3.31	-5.56 +/- 0.10	-68.56 +/- 3.67	-29.89 +/- 1.86
Compound (S*a*)-3	**εprot=2 εsol=80**	-50.63 +/- 1.28	-22.59 +/- 2.0	-54.58 +/- 2.73	-42.36 +/- 4.33	-5.33 +/- 0.10	-61.03 +/- 4.67	-23.98 +/- 1.61
Compound (Sa)-5	**εprot=2 εsol=80**	- 56.57+/- 1.90	-11.33 +/- 2.44	43.69 +/- 2.38	-41.96 +/- 3.67	-5.64 +/- 0.15	-66.17 +/- 3.62	-29.86 +/- 1.85
Compound 6	**εprot=2 εsol=80**	-40.88 +/- 0.93	-7.14 +/- 0.74	24.78 +/- 1.04	-28.25 +/- 3.84	-4.10 +/- 0.08	-51.53 +/- 4.07	-27.35 +/- 1.19
Compound 1	**εprot=2 εsol=80**	-48.37 +/- 1.32	77.69 +/- 3.05	38.31 +/- 1.82	-35.72 +/- 4.16	-5.27 +/- 0.12	-27.27 +/- 5.54	3.23 +/- 2.67
Compound 7	**εprot=2 εsol=80**	-39.31 +/- 1.67	-4.44 +/- 1.01	21.83 +/- 1.11	-31.99 +/- 3.24	-4.31 +/- 0.14	-53.90 +/- 3.53	-26.22 +/- 1.04
Compound 8	**εprot=2 εsol=80**	-41.93 +/- 1.05	-2.73 +/- 0.60	26.2 +/- 1.36	-34.1 +/- 2.79	-4.63 +/- 0.08	-52.56 +/- 3.09	-23.08 +/- 1.02
Compound 11	**εprot=2 εsol=80**	-39.27 +/- 1.17	-2.17 +/- 1.18	24.05 +/- 1.21	-32.39 +/- 2.11	-4.32 +/- 0.09	-49.78 +/- 2.42	-21.70 +/- 1.20
Compound 9	**εprot=2 εsol=80**	-39.18 +/- 1.47	-5.23 +/- 1.23	29.84 +/- 2.02	-32.87 +/- 1.49	-4.58 +/- 0.11	-47.44 +/- 2.72	-19.17 +/- 1.38
Compound 10	**εprot=2 εsol=80**	-31.17 +/- 1.07	0.02 +/- 0.58	17.89 +/- 1.54	-25.32 +/- 2.22	-3.93 +/- 0.09	-38.57 +/- 2.45	-17.18 +/- 1.13

Supplementary table 4: Calculated MM-PBSA individual interaction energy of residues within 10 Å from ligand in the PI3Kα (*Sa*)-2 complex (4L23) using internal and solvent dielectric constant set to 2 and 80, respectively.

Residue	Energy (kcal/mol)	Distance (Å)	Residue	Energy (kcal/mol)	Distance (Å)	Residue	Energy (kcal/mol)	Distance (Å)
Gln728	0.00	9	Asn803	-0.01	9	His855	-0.17	2.5
Leu755	-0.01	10	Asp805	-0.09	0	Thr856	-1.10	3
Glu768	-0.14	9.5	Asp806	-0.04	9	Ile857	-0.07	0
Arg770	0.53	5	Leu807	-0.03	6	Met858	-0.17	5.5
Ile771	-0.03	8.5	Gln809	0.01	10	Gln859	0.76	3.5
Met772	-1.45	2.5	Asp810	0.26	6	Ile860	-0.05	0
Ser773	-0.10	5.5	Leu814	-0.02	0	Gln861	-0.02	10
Ser774	-0.12	3.5	Leu834	-0.01	8.5	Cys862	-0.02	9.5
Ala775	-0.05	5.5	Tyr836	-0.02	5.5	Lys863	0.07	8.5
Lys776	-0.29	3.5	Gly837	0.03	8.5	Trp880	-0.02	9
Arg777	0.09	6.5	Cys838	-0.01	8.5	Gly914	-0.02	10
Pro778	-0.52	3	Gly846	-0.04	9	Asp915	-0.05	0
Leu779	-0.08	0	Leu847	-0.01	7.5	Arg916	-0.38	0
Trp780	-1.64	3	Ile848	-0.71	3	His917	0.10	3
Leu781	-0.02	9.5	Glu849	0.45	4.5	Asn918	-0.05	0
Glu798	-0.20	0	Val850	-1.21	2.5	Ser919	0.55	3
Ile799	-0.08	7.5	Val851	-0.31	2	Asn920	-0.72	3.5
Ile800	-1.58	3	Arg852	-0.15	5	Ile921	-0.05	0
Phe801	-0.08	0	Asn853	-0.08	6	Met922	-1.15	2.5
Lys802	0.47	2.5	Ser854	0.33	3	Val923	-0.02	6.5
Gln728	0.00	9	Asn803	-0.01	9	Lys924	-0.07	7.5
Leu755	-0.01	10	Asp805	-0.09	0	Asp925	0.00	9
Glu768	-0.14	9.5	Asp806	-0.04	9	Phe930	-0.21	4.5
Arg770	0.53	5	Leu807	-0.03	6	His931	-0.14	6.5
Ile771	-0.03	8.5	Gln809	0.01	10	Ile932	-2.79	3
Met772	-1.45	2.5	Asp810	0.26	6	Asp933	2.89	2.5
Ser773	-0.10	5.5	Leu814	-0.02	0			
Ser774	-0.12	3.5	Leu834	-0.01	8.5			

Supplementary table 5: Calculated MM-PBSA individual interaction energy of residues within 10 Å from ligand in the PI3Kα (*Ra*)-2complex (4YKN) using internal and solvent dielectric constant set to 2 and 80, respectively.

**Residue**	**Energy (kJ/mol)**	**Distance (Å)**	**Residue**	**Energy (kJ/mol)**	**Distance (Å)**	**Residue**	**Energy (kJ/mol)**	**Distance (Å)**
Glu849	-0.16	2	Leu814	-0.03	5.5	Val923	-0.02	8
Lys776	0.14	2.5	Arg852	0.12	5.5	Ile771	-0.02	8.5
Val850	-0.99	2.5	Ser773	-0.15	6	Asn803	-0.03	8.5
Val851	-0.41	2.5	Asp805	-0.13	6	Met811	-0.01	8.5
Asp933	0.43	2.5	Gly837	0.03	6	Thr813	-0.04	8.5
Ser774	-0.19	3	His855	-0.05	6	Gly914	0.00	8.5
Ala775	-0.04	3	Ile921	-0.07	6	Arg770	0.50	9
Pro778	-0.99	3	Gly935	-0.07	6	Gln809	-0.02	9
Tyr836	-1.07	3	Ile799	-0.11	6.5	Tyr904	-0.02	9
Ile848	-1.66	3	Cys838	-0.05	6.5	Leu839	-0.02	9.5
Thr856	-0.64	3	Asn853	-0.02	6.5	Gly846	-0.05	9.5
Asn920	-0.29	3	Arg916	-0.17	6.5	Ile913	-0.02	9.5
Met922	-0.86	3	His931	-0.05	6.5	Gln728	0.02	10
Met772	-1.65	3.5	Leu847	-0.01	7	Arg808	0.01	10
Ile932	-3.34	3.5	Met858	-0.07	7	Ile860	0.00	10
Ile800	-1.53	4	His917	0.00	7	Leu870	0.00	10
Asp810	0.79	4	Asn918	-0.07	7	Asp925	-0.07	10
Ser854	0.02	4	Leu755	-0.02	7.5			
Ser919	0.50	4	Asp806	-0.10	7.5			
Arg777	0.28	4.5	Leu834	0.01	7.5			
Trp780	-1.05	4.5	Pro835	-0.11	7.5			
Phe930	-0.19	4.5	Ile857	-0.05	7.5			
Phe934	-0.17	4.5	Gln859	0.00	7.5			
Lys802	0.64	5	Lys924	0.14	7.5			
Asp915	-0.17	5	Leu779	-0.10	8			
His936	-0.14	5	Glu798	-0.44	8			
Leu807	-0.04	5.5	Phe801	-0.14	8			

Supplementary table 6: Calculated MM-PBSA individual interaction energy of residues within 10 Å from ligand in the PI3Kα (*Ra*)-3 complex (4YKN) using internal and solvent dielectric constant set to 2 and 80, respectively.

Residue	Energy (kcal/mol)	Distance (Å)	Residue	Energy (kcal/mol)	Distance (Å)	Residue	Energy (kcal/mol)	Distance (Å)
Asp805	-0.57	2	Cys838	-0.05	5.5	Ile921	-0.07	8
Glu849	-0.14	2	Gly804	0.01	6	Gln728	-0.02	8.5
Met772	-1.02	2.5	Leu814	-0.07	6	Met811	-0.01	8.5
Pro778	-1.08	2.5	His855	-0.07	6	Thr813	-0.01	8.5
Ile800	-2.05	2.5	His917	-0.02	6	Pro835	-0.06	8.5
Tyr836	-0.48	2.5	Ser919	0.41	6	Val923	-0.02	8.5
Val851	-0.41	2.5	Asn920	-0.19	6	Gln809	-0.03	9
Ser774	-0.04	3	Phe934	-0.14	6	Leu839	-0.02	9
Lys776	0.34	3	Arg852	0.02	6.5	Gly846	-0.05	9
Arg777	0.02	3	Gly935	-0.05	6.5	Ile857	-0.02	9
Trp780	-1.23	3	Leu755	-0.02	7	Asn918	-0.05	9
Lys802	0.81	3	Ile799	-0.13	7	Lys924	0.12	9
Ile848	-1.79	3	Asn803	-0.03	7	Ile817	-0.01	9.5
Met922	-0.76	3	His931	-0.10	7	Val845	0.01	9.5
Ala775	-0.02	3.5	Arg770	0.39	7.5	Leu781	-0.03	10
Val850	-1.58	3.5	Ile771	-0.06	7.5	Tyr904	-0.02	10
Ile932	-3.27	3.5	Leu779	-0.10	7.5			
Asp933	0.76	3.5	Phe801	-0.09	7.5			
Asp810	0.64	4	Leu834	-0.02	7.5			
Thr856	-0.41	4.5	Gln859	-0.02	7.5			
Ser773	-0.03	5	Arg916	0.00	7.5			
Leu807	-0.08	5	Glu798	-0.40	8			
Ser854	-0.14	5	Cys844	-0.02	8			
Phe930	-0.29	5	Leu847	-0.06	8			
Asp806	-0.14	5.5	Asn853	-0.05	8			
Gly837	0.02	5.5	Met858	-0.02	8			

Supplementary table 7: Calculated MM-PBSA individual interaction energy of residues within 10 Å from ligand in the PI3Kα (*Sa*)-4 complex (4YKN) using internal and solvent dielectric constant set to 2 and 80, respectively.

Residue	Energy (kJ/mol)	Distance (Å)	Residue	Energy (kJ/mol)	Distance (Å)	Residue	Energy (kJ/mol)	Distance (Å)
Met772	-1.65	2.5	Asn920	-0.41	5.5	Arg852	0.01	7.5
Ser774	-0.20	2.5	Phe930	-0.24	5.5	Lys863	0.14	7.5
Thr856	-0.76	2.5	Ala775	-0.05	6	Phe872	0.00	7.5
Gln859	0.10	2.5	Asp805	-0.35	6	Glu798	-0.40	8
Ser919	0.69	2.5	Cys862	-0.02	6	Asp806	-0.09	8
Met922	-0.72	2.5	His917	0.19	6	Gly837	0.15	8
Asp933	3.84	2.5	Arg770	0.43	6.5	Gln861	-0.02	8
Pro778	-0.54	3	Ile771	0.01	6.5	Lys924	-0.26	8
Trp780	-1.08	3	Leu779	-0.08	6.5	Leu847	-0.01	8.5
Ile800	-1.86	3	Leu807	-0.03	6.5	Gly935	-0.10	8.5
Ile848	-1.29	3	Ile857	-0.10	6.5	Asn803	-0.02	9
Val850	-0.91	3	Asp810	1.82	7	Asp915	0.21	9
Ser854	-0.11	3	Asn853	-0.01	7	Asp925	-0.05	9
His855	-0.09	3	Ile860	-0.05	7	Leu755	-0.01	9.5
Met858	-0.50	3	Arg916	-0.48	7	Leu781	-0.02	9.5
Ile932	-3.03	3.5	Ile921	-0.05	7	Leu834	0.04	9.5
Ser773	-0.10	4	Val923	-0.05	7	Gly846	-0.05	9.5
Lys776	0.09	4	His931	0.00	7	Trp880	0.00	9.5
Lys802	1.28	4	Phe934	-0.12	7			
Val851	-0.27	4.5	Gln728	0.01	7.5			
Arg777	0.24	5	Ile799	-0.14	7.5			
Glu849	1.21	5	Phe801	-0.11	7.5			
Tyr836	-0.28	5.5	Leu814	-0.03	7.5			
Asn918	-0.02	5.5	Cys838	0.00	7.5			

Supplementary table 8: Calculated MM-PBSA individual interaction energy of residues within 10 Å from ligand in the PI3Kα (*Ra*)-4 complex (4YKN) using internal and solvent dielectric constant set to 2 and 80, respectively.

Residue	Energy (kJ/mol)	Distance (Å)	Residue	Energy (kJ/mol)	Distance (Å)	Residue	Energy (kJ/mol)	Distance (Å)
Val1851	-0.2	2	Arg1852	0.1	5	Lys1924	-0.1	8
Ile1932	-2.8	2.5	Leu1814	-0.06	5.5	Ile1860	-0.1	8
Met1772	-2.08	2.5	Gly1935	0.0	5.5	Gly1846	-0.03	8
Val1850	-1.4	2.5	Asp1810	0.62	5.5	Gln1728	0.00	8
Trp1780	-1.39	2.5	Arg1770	1.19	5.5	Leu1870	0.0	8
Met1922	-1.0	2.5	Phe1801	-0.09	6	Leu1834	0.04	8
Pro1778	-0.58	2.5	Asp1806	0.09	6	Ile1771	-0.02	8.5
Lys1802	-0.19	2.5	His1931	-0.1	6.5	Pro1835	-0.10	9
His1917	0.2	2.5	Ile1921	-0.1	6.5	Cys1862	0.0	9
Ile1800	-1.38	3	Cys1838	-0.05	6.5	Gln1809	0.00	9
Ile1848	-0.99	3	Asn1853	0.0	6.5	Gly1914	0.0	9
Thr1856	-0.7	3	Leu1779	-0.03	6.5	Leu1781	-0.01	9.5
Tyr1836	-0.15	3	Asp1915	0.1	6.5	Gly1804	0.00	9.5
Ser1919	0.5	3	Arg1777	0.22	6.5	Thr1716	0.00	9.5
Glu1849	1.2	3	Gly1837	0.23	6.5	Met1811	0.00	9.5
Asp1933	2.7	3	Arg1916	-0.5	7	Leu1839	0.00	9.5
Ser1774	-0.25	3.5	Met1858	-0.3	7	Lys1863	0.2	9.5
Ser1854	0.3	3.5	Phe1934	-0.1	7	Arg1808	-0.07	10
Gln1859	0.4	3.5	Asn1918	-0.1	7	Tyr1904	0.0	10
Asp1805	0.00	4	Ile1857	-0.1	7	Ile1913	0.0	10
Phe1930	-0.3	4.5	Val1923	0.0	7	Cys1844	-0.01	10
Asn1920	-0.2	4.5	Glu1798	-0.32	7.5			
His1855	-0.1	4.5	Ile1799	-0.15	7.5			
Ser1773	-0.26	5	Ala1775	-0.04	7.5			
Leu1807	-0.01	5	Leu1847	-0.02	7.5			
His1936	0.0	5	Leu1755	-0.01	7.5			
Lys1776	0.04	5	Asn1803	-0.01	7.5			

Supplementary table 9: Calculated MM-PBSA individual interaction energy of residues within 10 Å from ligand in the PI3Kα (*Ra*)-2 complex (4YKN) using internal and solvent dielectric constant set to 2 and 80, respectively.

Residue	Energy (kJ/mol)	Distance (Å)	Residue	Energy (kJ/mol)	Distance (Å)	Residue	Energy (kJ/mol)	Distance (Å)
Met772	-1.84	2.5	Ile771	0.02	5	Val845	0.02	9
Ser773	-0.07	2.5	Ala775	-0.10	5	Leu755	-0.02	9.5
Ile800	-1.69	2.5	Asn803	-0.01	5	Cys769	0.01	9.5
Ile848	-1.05	2.5	Leu847	-0.04	5	Arg808	-0.02	9.5
Met922	-0.95	2.5	Asp810	1.55	5.5	Gln809	-0.10	9.5
Ser774	-0.46	3	Arg852	-0.36	5.5	Pro835	-0.04	9.5
Pro778	-0.90	3	His855	-0.03	5.5	Ser840	0.01	9.5
Trp780	-1.23	3	Gln728	-0.02	6	Ile860	-0.05	9.5
Lys802	0.41	3	Lys776	-0.13	6	Phe872	-0.01	9.5
Val851	-0.13	3	Leu779	-0.10	6	Asp925	0.24	9.5
His917	0.19	3	Gly846	-0.09	6	Thr716	0.00	10
Ser919	0.82	3	Asp915	0.39	6	Met732	-0.01	10
Ile932	-2.37	3	Asp805	-0.19	6.5	Cys844	-0.01	10
Asp933	3.85	3	Cys838	-0.03	6.5	Gln861	-0.05	10
Val850	-0.86	3.5	Phe934	-0.15	6.5	Gly914	0.05	10
Leu807	-0.04	4	Glu798	0.31	7.5			
Tyr836	-0.09	4	Ile799	-0.12	7.5			
Glu849	1.49	4	Leu834	0.02	7.5			
Ser854	0.21	4	Gly837	0.14	7.5			
Thr856	-0.97	4	Cys862	-0.06	7.5			
Gln859	-0.10	4	Lys924	-0.33	7.5			
Asn920	-0.36	4	Gly804	0.00	8.5			
Arg770	0.06	4.5	Met811	0.00	8.5			
Phe801	-0.13	4.5	Ile857	-0.11	8.5			
Met858	-0.51	4.5	His936	-0.03	8.5			
Phe930	-0.23	4.5	Glu768	0.15	9			
			Leu781	-0.01	9			

Supplementary table 10: Calculated MM-PBSA individual interaction energy of residues within 10 Å from ligand in the mTOR (*Sa*)-5 complex (4SX) using internal and solvent dielectric constant set to 2 and 80, respectively.

Residue	Energy (kJ/mol)	Distance (Å)	Residue	Energy (kJ/mol)	Distance (Å)	Residue	Energy (kJ/mol)	Distance (Å)
Glu810	1.55	2	Hie809	0.14	5	Leu856	-0.37	8
Ash815	2.28	2	Pro861	-0.02	5	Arg871	-1.55	8
Gln787	1.21	2.5	Ser962	0.32	5	Asp967	1.26	8
Leu805	-8.97	2.5	Arg788	-0.54	5	Arg1050	-0.42	8
Lys807	11.56	2.5	Met819	-0.43	5.5	Val782	0.04	8.5
Tyr845	-1.27	2.5	Hid862	-0.37	5.5	Val818	-0.36	8.5
Ile857	-8.57	2.5	Hid867	-0.46	5.5	Phe822	-0.34	8.5
Val860	-1.04	2.5	Asn963	0.10	6	Gln843	-0.17	8.5
Asp864	1.57	2.5	Leu966	-0.02	6	Ile848	-0.17	8.5
Ile976	-13.2	2.5	Val803	-0.14	6	Gly855	-0.28	8.5
Ile783	-5.65	3	Asp811	-0.19	6	Hid960	0.19	8.5
Pro789	-2.43	3	Ala846	0.41	6	Pro961	0.10	8.5
Gly858	4.77	3	Leu964	-0.32	6.5	Ser854	0.20	9
Trp859	-11.1	3	Gly979	-0.31	6.5	Ile870	-0.12	9
Thr865	-4.31	3	Thr784	-0.20	6.5	Asp980	0.79	9
Met965	-5.55	3	Lys786	-0.26	7.5	Gln781	-0.25	9.5
Asp977	3.87	3	Gln814	-0.06	7.5	Leu792	-0.02	9.5
Ser785	-0.19	3.5	Arg844	-1.20	7.5	Arg813	-0.30	9.5
Cys863	-0.69	3.5	Leu869	-0.24	7.5	Asn853	-0.03	9.5
Ala868	-0.63	4	Arg790	-0.59	7.5	Lys926	-0.35	9.5
Arg968	-1.66	4	Phe804	-0.71	7.5	Leu956	-0.10	9.5
Phe978	-1.99	4.5	Leu806	-0.59	8.5	Asp958	0.81	10
Leu812	-0.81	5	Gly808	-0.01	8.5	Ile973	-0.19	10
Val847	-0.18	5	Leu866	-0.36	8.5	Gly762	-0.03	10
Leu974	-2.73	5	Asp872	0.28	8.5			
Hid975	-0.53	5	Arg959	-1.82	9			
Lys791	-2.14	5.5	Glu816	0.54	9			

Supplementary table 11: Calculated MM-PBSA individual interaction energy of residues within 10 Å from ligand in the mTOR(*Sa*)-2 complex (4SX) using internal and solvent dielectric constant set to 2 and 80, respectively.

Residue	Energy (kJ/mol)	Distance (Å)	Residue	Energy (kJ/mol)	Distance (Å)	Residue	Energy (kJ/mol)	Distance (Å)
Asp977	9.22	2	Pro861	-0.10	5	Gln814	-0.08	8
Ile783	-6.82	2.5	Ser785	0.35	5	Gly808	-0.03	8
Lys807	1.42	2.5	Thr784	-0.29	5	Gly979	-0.08	8
Trp859	-8.92	2.5	Arg959	-0.98	5.5	Ile848	-0.12	8
Val860	-2.33	2.5	Hie809	-0.08	5.5	Leu869	-0.13	8
Asn963	-1.14	3	Hid862	-0.22	6	Arg813	-0.05	8.5
Glu810	1.94	3	Met819	-0.02	6	Asp980	-0.87	8.5
Ile857	-6.86	3	Ala846	-0.12	6.5	Glu816	-0.18	8.5
Ile976	-12.9	3	Asp811	-0.18	6.5	Leu792	-0.40	8.5
Thr865	-1.92	3	Leu964	-0.18	6.5	Phe822	-0.11	8.5
Tyr845	-2.43	3	Leu974	-0.41	6.5	Asn853	-0.04	9
Ash815	0.48	3.5	Lys786	0.17	6.5	Gln843	-0.21	9
Gly858	2.5	3.5	Lys791	-2.74	6.5	Gly957	0.01	9
Leu805	-13.87	3.5	Phe804	-3.54	6.5	Val818	0.10	9
Met965	-2.30	3.5	Val803	-2.26	6.5	Arg871	-0.26	9.5
Pro789	-2.28	3.5	Arg968	-1.07	7	Asp967	0.69	9.5
Ser962	1.47	3.5	Asp958	-0.92	7	Leu956	-0.05	9.5
Cys863	-0.07	4	Gly855	-0.09	7	Met947	-0.08	9.5
Leu812	-0.90	4	Val782	-0.14	7	Ser854	0.01	9.5
Asp864	0.78	4.5	Arg844	-0.42	7.5	Ile870	-0.05	10
Gln787	1.20	4.5	Leu806	-0.53	7.5	Pro849	0.02	10
Hid960	0.88	4.5	Leu856	-0.67	7.5	Thr793	-0.18	10
Phe978	-0.99	4.5	Leu866	-0.14	7.5			
Phe978	-0.99	4.5	Leu966	0.02	7.5			
Val847	-0.47	4.5	Pro961	-0.01	7.5			
Ala868	-0.29	5	Arg788	0.13	8			
Hid867	-0.38	5	Arg790	-0.48	8			

Supplementary table 12: Calculated MM-PBSA individual interaction energy of residues within 10 Å from ligand in the mTOR (*Sa*)-4 complex (4SX) using internal and solvent dielectric constant set to 2 and 80, respectively.

Residue	Energy (kJ/mol)	Distance (Å)	Residue	Energy (kJ/mol)	Distance (Å)	Residue	Energy (kJ/mol)	Distance (Å)
Ile2163	-12.40	2.5	Asp2244	7.85	5	Met2199	-0.09	7.5
Ser2165	-0.93	2.5	Hid2340	2.3	5	Gln2223	0.06	7.5
Gln2167	-0.28	2.5	Leu2354	-0.3	5	Val2227	-0.07	7.5
Lys2187	-2.27	2.5	Tyr2542	-0.1	5	Leu2246	-0.5	7.5
Val2240	-0.43	2.5	Arg2168	-1.10	5.5	Asp2347	1.9	7.5
Met2345	-5.2	2.5	Glu2190	0.60	5.5	Leu2186	-0.18	8
Asp2357	6.1	2.5	Hid2265	0.0	5.5	Gly2188	-0.05	8
Leu2185	-5.62	3	Pro2341	-0.1	5.5	Ala2226	0.19	8
Ile2237	-0.52	3	Asn2343	-1.2	5.5	Leu2236	-0.15	8
Gly2238	1.52	3	Phe2358	-0.1	5.5	Arg2339	-4.0	8
Trp2239	-12.60	3	Cys2546	0.1	5.5	Leu2344	0.2	8
Cys2243	4.85	3	Hid2242	-0.90	6	Gly2359	0.0	8
Hid2247	-3.5	3	Trp2549	1.3	6	Phe2548	-0.1	8
Ala2248	-3.1	3	Phe2184	-0.45	6.5	Leu2172	0.01	8.5
Ser2342	1.1	3	Leu2249	-0.7	6.5	Arg2224	-0.81	8.5
Ile2356	-9.5	3	Ile2250	-0.3	6.5	Arg2254	-1.2	8.5
Pro2169	-1.55	3.5	Arg2348	-2.5	6.5	Asn2262	-0.1	8.5
Thr2245	-2.17	3.5	Hid2355	-0.6	6.5	Hie2189	-0.01	9
Thr2164	1.01	4	Ile2543	0.1	6.5	Gly2235	-0.02	9
Arg2251	-0.4	4	Trp2545	0.0	6.5	Glu2255	0.2	9
Gly2544	0.1	4	Arg2170	-1.19	7	Asp2338	3.2	9
Leu2261	-0.2	4.5	Val2183	-0.57	7	Asp2360	1.1	9
Gln2161	0.45	5	Leu2192	-0.02	7	Arg2378	-2.0	9
Lys2166	-1.34	5	Asp2252	-0.1	7	Phe2202	-0.08	9.5
Lys2171	-2.63	5	Leu2346	0.3	7	Tyr2253	-0.1	9.5
Tyr2225	-0.19	5	Val2162	-0.38	7.5	Glu2264	1.0	10
Pro2241	-0.14	5	Ash2195	0.13	7.5	Lys2352	-1.0	10

Supplementary table 13: Calculated MM-PBSA individual interaction energy of residues within 10 Å from ligand in the mTOR(*Ra*)-3 complex (4SX) using internal and solvent dielectric constant set to 2 and 80, respectively.

Residue	Energy (kJ/mol)	Distance (Å)	Residue	Energy (kJ/mol)	Distance (Å)	Residue	Energy (kJ/mol)	Distance (Å)
Asn2343	-0.5	2	Pro2241	-0.16	5.5	Leu2236	0.02	8
Asp2357	10.3	2	Arg2339	-5.5	5.5	Leu2246	-0.6	8
Ile2163	-9.00	2.5	Arg2348	-3.1	5.5	Hid2247	-1.1	8.5
Lys2187	-2.24	2.5	Asp2360	10.8	5.5	Leu2249	-0.3	8.5
Ile2237	-1.25	2.5	Hid2242	-0.6	6	Gly2337	0.4	8.5
Trp2239	-15.09	2.5	Gly2359	-0.9	6	Arg2368	-2.3	8.5
Val2240	-1.39	2.5	Lys2166	-5.12	6.5	Gln2223	-0.12	9
Pro2169	-2.69	3	Arg2168	0.50	6.5	Leu2336	-0.2	9
Leu2185	-7.20	3	Val2183	-0.31	6.5	Leu2172	-0.04	9.5
Cys2243	0.8	3	Val2227	0.07	6.5	Asp211	0.21	9.5
Asp224	3.6	3	Leu2344	0.2	6.5	Phe2202	-0.07	9.5
Thr2245	-4.7	3	Phe2358	-0.6	6.5	Arg224	-0.46	9.5
Met235	-4.2	3	Phe2184	-0.11	7	Ile2228	-0.07	9.5
Ile2356	-12.5	3	Leu2186	-0.49	7	Ser2234	0.05	9.5
Ser2342	1.2	3.5	Leu2192	-0.05	7			
Ser2165	0.26	4	Ala2248	-0.7	7			
Gln2161	-0.24	4.5	Leu2346	0.2	7			
Gln2167	0.98	4.5	Hid2355	-1.0	7			
Glu2190	1.12	4.5	Val2162	-0.05	7.5			
Tyr2225	-0.81	4.5	Arg2170	-0.19	7.5			
Gly2238	-0.51	4.5	Gly2188	0.00	7.5			
Leu2354	-0.1	4.5	Hie2189	-0.25	7.5			
Thr2164	1.87	5	Met2199	-0.06	7.5			
Lys2171	-2.31	5	Ala2226	-0.19	7.5			
Asp2338	12.7	5	Pro2341	-0.1	7.5			
Hid2340	4.0	5	Asp2347	1.7	7.5			
Ash2195	-0.07	5.5	Gly2235	-0.12	8			

Supplementary table 14: Calculated MM-PBSA individual interaction energy of residues within 10 Å from ligand in the mTOR *(Sa)*-3 complex (4SX) using internal and solvent dielectric constant set to 2 and 80, respectively.

Residue	Energy (kJ/mol)	Distance (Å)	Residue	Energy (kJ/mol)	Distance (Å)	Residue	Energy (kJ/mol)	Distance (Å)
Lys2187	-1.59	2.5	His2247	-0.2	6	Ile2250	-0.1	8
Val2240	-0.19	2.5	Leu2249	-0.3	6	His2340	-0.1	8
Asp2244	8.52	2.5	His2355	-0.4	6	Pro2341	0.0	8
Thr2245	-2.95	2.5	Gly2359	-0.2	6	Leu2344	-0.3	8
Asp2357	0.7	2.5	Arg2168	-0.23	6.5	Gly2235	-0.18	8.5
Pro2169	-1.91	3	Arg2170	-0.38	6.5	Lys2306	-0.1	8.5
Tyr2225	-3.22	3	Leu2186	-1.17	6.5	Asp2347	0.8	8.5
Cys2243	3.0	3	Gly2188	-0.06	6.5	Asp2360	0.3	8.5
Arg2251	-1.3	3	Val2227	-0.79	6.5	Arg2193	-0.32	9
Leu2185	-10.10	3.5	His2242	-0.4	6.5	Gln2194	-0.12	9
Glu2190	7.82	3.5	Leu2346	0.1	6.5	Glu2196	-0.35	9
Trp2239	-7.25	3.5	Lys2166	-0.44	7	Asp2338	0.6	9
Ala2248	-1.9	3.5	Lys2171	0.00	7	Leu2172	-0.11	9.5
Met2345	-4.0	3.5	Gln2223	-0.04	7	Val2198	-0.09	9.5
Ile2356	-11.7	3.5	Leu2246	-0.25	7	Ile2228	-0.23	9.5
Ile2163	-2.10	4	Asp2252	-0.1	7	Asn2233	-0.02	9.5
Ile2237	-10.53	4	Arg2339	-0.9	7	Phe2202	-0.08	10
Gly2238	3.21	4	Thr2164	-0.06	7.5	Ser2234	0.00	10
Ser2165	-0.71	4.5	Phe2184	-0.38	7.5	Tyr2253	0.0	10
Gln2167	-0.50	4.5	Asp2191	0.91	7.5	Leu2336	-0.1	10
Leu2192	-3.32	4.5	Met2199	-0.15	7.5	Cys2361	0.0	10
Asp2195	0.58	4.5	Val2162	0.04	8			
Pro2241	0.0	4.5	Val2183	-0.10	8			
Phe2358	-1.3	4.5	His2189	-0.01	8			
Asn2343	-0.8	5	Arg2224	-0.38	8			
Arg2348	-2.3	5	Ala2226	-0.34	8			
Leu2354	-0.8	5	Leu2236	-0.55	8			

Supplementary table 15: Calculated MM-PBSA individual interaction energy of residues within 10 Å from ligand in the mTOR (*Ra*)-4 complex (4SX) using internal and solvent dielectric constant set to 2 and 80, respectively.

Residue	Energy (kJ/mol)	Distance (Å)	Residue	Energy (kJ/mol)	Distance (Å)	Residue	Energy (kJ/mol)	Distance (Å)
Gln2167	-0.20	2.5	asp2191	-0.15	6	Val2183	-0.27	8.5
Leu2185	-7.22	3	Lys2171	-2.24	6.5	Gly2188	0.04	8.5
Glu2190	0.48	3	Phe2184	-0.28	6.5	Leu2246	-0.58	8.5
His2340	3.43	3	His2189	0.03	6.5	His2247	-1.27	8.5
Asn2343	-3.79	3	Pro2241	0.00	6.5	Leu2346	0.24	8.5
Asp2357	11.80	3	Asp2244	9.31	6.5	Arg2348	-4.85	8.5
Asp2195	-0.25	3.5	His2355	-1.21	6.5	Gly2544	0.21	8.5
Tyr2225	-1.02	3.5	Lys2166	-1.09	7	Ile2228	-0.06	9
Ile2237	-1.71	3.5	Leu2186	-0.49	7	Asn2233	-0.05	9
Gly2238	1.07	3.5	Gln2223	-0.19	7	Gly2235	-0.13	9
Trp2239	-15.55	3.5	Val2227	0.02	7	Leu2249	-0.50	9
Val2240	-1.31	3.5	Ala2248	-2.03	7	Asp2347	2.04	9
Ser2342	1.41	3.5	Pro2341	-0.07	7	Cys2361	-0.05	9
Met2345	-4.39	3.5	Leu2344	0.06	7	Val2198	0.00	9.5
Ile2356	-11.70	3.5	Gln2161	-0.21	7.5	Ser2234	0.07	9.5
Ile2163	-10.55	4	Thr2164	1.14	7.5	Gly2337	0.25	9.5
Pro2169	-2.76	4	Arg2168	-0.42	7.5	Ile2353	-0.24	9.5
Lys2187	2.70	4	Leu2172	0.00	7.5	Arg2368	-1.09	9.5
Leu2192	-0.06	4	Gln2194	0.10	7.5	Trp2429	-0.02	9.5
Leu2354	-0.20	4	Met2199	0.05	7.5	Tyr2542	-0.06	9.5
Phe2358	-0.33	4	Arg2224	-0.74	7.5	Trp2545	-0.03	9.5
Ser2165	-1.11	4.5	Ala2226	0.00	7.5	Thr2232	0.00	10
Thr2245	-3.59	4.5	Leu2236	-0.10	7.5	Leu2336	-0.13	10
Asp2338	4.20	5	Arg2339	-4.74	7.5			
Gly2359	-0.70	5	Asp2360	3.4	7.5			
Cys2243	1.38	5.5	Val2162	-0.08	8			
Arg2170	-0.67	6	Arg2193	0.02	8			

Supplementary table 16: Pharmacokinetic properties (physicochemical properties, lipophilicity, water solubility, drug-likeness, and medicinal chemistry) of the four selected compounds.

Properties	Parameters	Compound 4	Compound 2	Compound 5	Compound 3
Physicochemical Properties	Molecular Weight	580.54	566.51	552.48	538.46
	Heavy Atoms	43	42	41	40
	Aromatic Atoms	32	32	32	32
	Rotatable Bonds	6	5	4	3
	H-Bond Acceptors	10	10	10	10
	H-Bond Donors	3	4	5	6
	Molar Refractivity	160.38	155.91	151.44	146.97
Lipophilicity	Log Po/w	6.04	5.74	5.44	5.13
Water Solubility	Log S (ESOL)	-7.39	-7.17	-6.96	-6.75
Pharmacokinetics	GI Absorption	Low	Low	Low	Low
Drug-likeness	Lipinski Violations	1	1	1	2
Medicinal Chemistry	Synthetic Accessibility	4.6	4.48	4.31	4.27

Supplementary table 17: Comparison between calculated MM/PBSA energies by sampling energies from the ranges 90-100 ns and 190-200 ns. Energies are given in $Kcal.mol^{-1}$

PI3Kα	90-100ns ΔG_{MMPBSA} (SASA)	190-200ns ΔG_{MMPBSA} (SASA)
Compound (*Sa*)-4	-22.82 +/-1.93	-30.25+/- 1.3
Compound (*Ra*)-4	-25.74 +/- 1.6	-28.35 +/-1.5
Compound (*Sa*)-2	-28.38 +/-1.5	-28.05 +/- 1.2
Compound (Ra)-2	-24.71 +/-1.8	-24.63+/- 1.6
Compound (Ra)-3	-28.28 +/-1.2	-28.35 +/- 1.2
Compound (Ra)-5	-30.66 +/- 1.5	-29.71+/- 1.5

mTOR	90-100ns ΔG_{MMPBSA} (SASA)	190-200ns ΔG_{MMPBSA} (SASA)
Compound (*Ra*)-4	-35.69 +/- 1.42	-36.35 +/- 1.5

Compound (S*a*)-4	-31.13 +/-2.09	-35.1+/- 1.2
Compound (Ra)-3	-29.89 +/-1.86	-29.72. +/- 1.6
Compound (S*a*)-3	-23.98 +/- 1.61	-21.90 +/- 1.5
Compound (*Sa*)-2	-32.32 +/-1.68	-28.05 +/- 1.2
Compound (Sa)-5	-29.86 +/-1.85	-35.11+/- 1.6
